# Inhaled nitric oxide as temporary respiratory stabilization in patients with COVID-19 related respiratory failure (INOCOV): Study protocol for a randomized controlled trial

**DOI:** 10.1371/journal.pone.0268822

**Published:** 2022-05-27

**Authors:** Jostein Skjalg Hagemo, Arne Kristian Skulberg, Marius Rehn, Morten Valberg, Maiju Pesonen, Hans Julius Heimdal, Fridtjof Heyerdahl

**Affiliations:** 1 Faculty of Medicine, Institute of Clinical Medicine, University of Oslo, Oslo, Norway; 2 Division of Prehospital Services, Air Ambulance Department, Oslo University Hospital, Oslo, Norway; 3 Department of Research, Norwegian Air Ambulance Foundation, Oslo, Norway; 4 Department of Circulation and Medical Imaging, Norwegian University of Science and Technology, Trondheim, Norway; 5 Faculty of Health Sciences, University of Stavanger, Stavanger, Norway; 6 Oslo Centre for Biostatistics and Epidemiology, Oslo University Hospital, Oslo, Norway; Universite de Bretagne Occidentale, FRANCE

## Abstract

**Background:**

In March 2020, WHO announced the COVID-19 a pandemic and a major global public health emergency. Mortality from COVID-19 is rapidly increasing globally, with acute respiratory failure as the predominant cause of death. Many patients experience severe hypoxia and life-threatening respiratory failure often requiring mechanical ventilation. To increase safety margins during emergency anaesthesia and rapid sequence intubation (RSI), patients are preoxygenated with a closed facemask with high-flow oxygen and positive end-expiratory pressure (PEEP). Due to the high shunt fraction of deoxygenated blood through the lungs frequently described in COVID-19 however, these measures may be insufficient to avoid harmful hypoxemia. Preoxygenation with inhaled nitric oxide (iNO) potentially reduces the shunt fraction and may thus allow for the necessary margins of safety during RSI.

**Methods and design:**

The INOCOV protocol describes a phase II pharmacological trial of inhaled nitric oxide (iNO) as an adjunct to standard of care with medical oxygen in initial airway and ventilation management of patients with known or suspected COVID-19 in acute respiratory failure. The trial is parallel two-arm, randomized, controlled, blinded trial. The primary outcome measure is the change in oxygen saturation (SpO_2_), and the null hypothesis is that there is no difference in the change in SpO_2_ following initiation of iNO.

**Trial registration:**

EudraCT number 2020-001656-18; WHO UTN: U1111-1250-1698.

**Protocol version:** 2.0 (June 25^th^, 2021).

## Background

The SARS-CoV-2 virus may cause a viral pneumonia with serious respiratory failure characterized by hypoxia, hypocapnia and elevated respiratory rate, defined as the Corona virus disease (COVID-19) [1]. Some patients deteriorate rapidly after a period with fewer and mild respiratory symptoms. Because many patients fail to recognize the severity of the hypoxia, the risk of rapid decompensation into life-threatening respiratory failure is evident. These patients often present with tachycardia and hypertension, being awake, but exhausted [2,3]. They respond variably to oxygen therapy indicating a need for respiratory support. Induction of anaesthesia and intubation in these hypoxic patients carries a high risk [4]. Initiatives to mitigate the risk of potentially life-threatening hypoxemia includes preoxygenation with high-flow oxygen, prone positioning and continuous positive airway pressure (CPAP). Due to atelectasis and an increased shunt fraction of deoxygenated blood, this may however be insufficient in patients with COVID-19 [4,5]. Inhaled nitric oxide (iNO) causes selective pulmonary vasodilation in ventilated segments and several studies find that iNO rapidly increases oxygen partial pressure in arterial blood in patients with adult respiratory distress syndrome (ARDS) [6–9]. This effect appears to be transient, lasting less than 72 hours. A recent systematic review concludes that there is currently insufficient evidence to support long-term iNO in hypoxic respiratory failure [6]. Nevertheless, iNO used as a temporary measure on certain indications is widespread in adult critical care [7,9–11]. During the current epidemic, iNO with doses starting at 20 parts per million (ppm) has successfully been used as rescue therapy to increase arterial oxygenation in patients with severe Covid-19 related ARDS [12–14].

We aim to perform a randomised trial to evaluate the effect of preoxygenation with 100% oxygen alone or in combination with iNO in hypoxic patients with suspected COVID-19, on the level of blood oxygen saturation (SpO_2_).

## Methods/Design

### Aim, design and setting

The protocol describes a parallel two-arm, blinded, randomized controlled phase II pharmacological trial with iNO as the investigational medical product (IMP) used as an adjunct to standard care in patients with known or suspected COVID-19 in acute respiratory failure.

The primary objective of the present trial is to evaluate the clinical efficacy of iNO to increase oxygen saturation prior to, during and after rapid sequence induction (RSI) emergency anaesthesia in patients with suspected or confirmed COVID-19 respiratory failure. Primary outcome measure is the change in SpO_2_ in the first five minutes after administration, and the null hypothesis is that there is no difference in the change in SpO_2_ following initiation of iNO and 100% oxygen compared to 100% oxygen alone. The trial is initiated as a single-centre study but is open for inclusion from other centres that have iNO delivered by INOblender® (INO Therapeutics LLC, Mallinckrodt Manufacturing LLC, Madison, Wisconsin, USA) as a treatment option.

**Fig 1:**
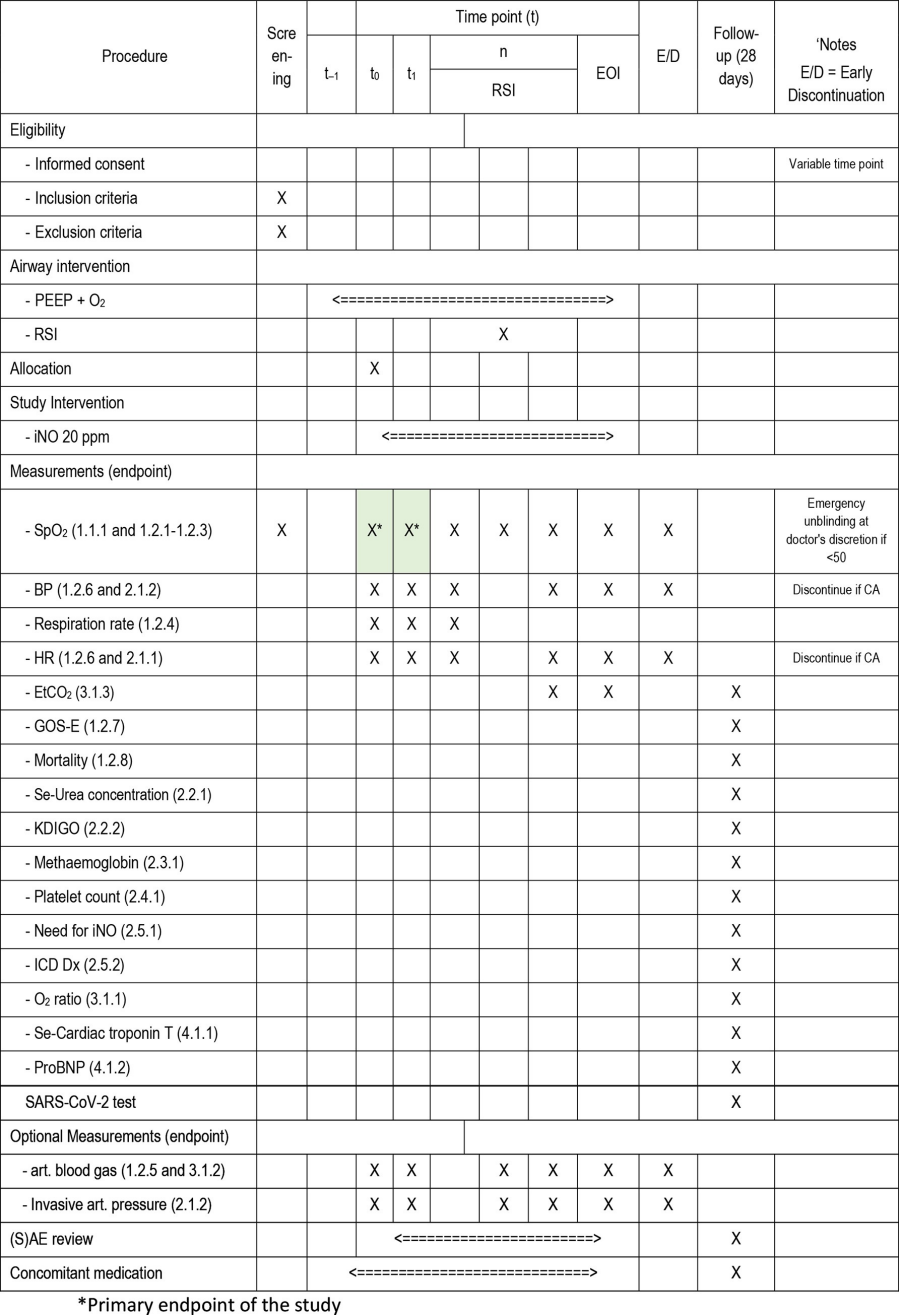
SPIRIT schedule.

The INOCOV protocol was designed using the Norwegian clinical research infrastructure network templates [15] and will be reported according to the Consolidated standards of reporting trials guidelines [16]. The protocol was prepared consulting the standard protocol items for clinical trials (SPIRIT) [17] (c.f. Fig 1 SPIRIT schedule and Additional file I SPIRIT checklists in S1 Checklist). The trial organisation was set up to fulfil the International conference on harmonization of technical requirements for registration of pharmaceuticals for human use (ICH) Good clinical practice (GCP) guideline. Trial registration: EudraCT number 2020-001656-18; WHO UTN: U1111-1250-1698. Given the current pandemic control and the following absence of subjects, formal agreement on funding from the Norwegian Air Ambulance Foundation has been postponed. The sponsor is the Air ambulance department at Oslo university hospital (OUH), Oslo, Norway. Regional Research Ethics Committee for Medicine and Health Services approved the study 18^th^ of May, 2020 (REK-128904).

### Characteristics of participants

Participants are recruited by emergency medical teams on-call. OUH, Air ambulance department crew consist of a consultant anaesthesiologist and helicopter emergency medical service (HEMS) crew or paramedic dispatched either by helicopter or rapid response car. To include patients in the present trial, the team members have to undergo formal training in pre-hospital iNO administration and the present study protocol.

### Inclusion criteria

- ≥18 years of age
- Suspected or probable cases of COVID-19 based on WHO definitions [18]
- Persistent SpO_2_ <90% despite three minutes preoxygenation with 10cm H_2_O positive end-expiratory pressure (PEEP) and high flow 100% oxygen

### Exclusion criteria

- Patients who after 3 mins of ventilation with 10cm H_2_O PEEP + 100% O_2_ i.e. at time of allocation (t0) have a SpO_2_ <50%, or a reliable SpO_2_ is impossible to obtain
- Patients in severe kidney failure or dialysis
- Known or suspected pregnancy based on information on the time of inclusion
- Hypersensitivity to the active substance (NO) or to the excipient (N_2_)
- Cardiac arrest
- Patient in prison or custody by police
- Staff present in treatment situation known to be pregnant
- Any reason why, in the opinion of the treating physician, it is probably in the best interest of the patient not to participate in the trial

### Informed consent procedure

The clinical setting in which the study is performed is by nature a situation where consent prior to inclusion and treatment is not possible to obtain. It would also be ethical questionable to trust that any information would be received and processed by the patient. Therefore, the study participants or next of kin will be informed and asked to participate post treatment. The informed consent procedure is as follows:

- Inclusion, treatment and data recording will occur before any informed consent is obtained
- After hospitalization, and within the 28 days to follow-up, either the patient (if deemed competent) or the next of kin will be informed with written information (see patient consent form) and asked to participate
- The consent will be for storing, analyzing and publish data collated in the CRF (treatment is already administered)
- If the consent is given by next of kind of a deceased patient, or a patient deemed to not recover to consent competency, the consent from next of kin is considered to be final
- If the consent is given by next of kin of a patient suspected to recover from incompetency, the consent from the patient will be obtained after recovery
- Information about the study and consent is given both oral (study personnel) and written (patient consent form)
- Consent is written (patient consent form). Oral consent can be obtained but require signature from two health care personnel (of which one study personnel/ investigator) stating that the patient has consented

### Randomization

Patients will be randomized in a 1:1 ratio between the two treatment arms, by a computer-generated block randomization with varying block sizes. Should more than one center be included, the randomization will be stratified by center. The institutional pharmacy at OUH will produce sealed envelopes with randomization, to be opened by the attending doctor at the time of inclusion.

### Blinding

The INOmax gas blender and cylinder is contained in a bag, and the cylinder valve with the pressure gauge will not be visible to the study doctor, only to the study assistant. The assistant will either open or mimic opening the INOmax cylinder according to allocation, and is instructed not to reveal the allocation, unless explicitly asked to do so in the case of emergency unblinding. The allocation will be unblinded after arrival in hospital, after the patient is intubated or a decision is made not to perform intubation.

### Intervention

**Fig 2:**
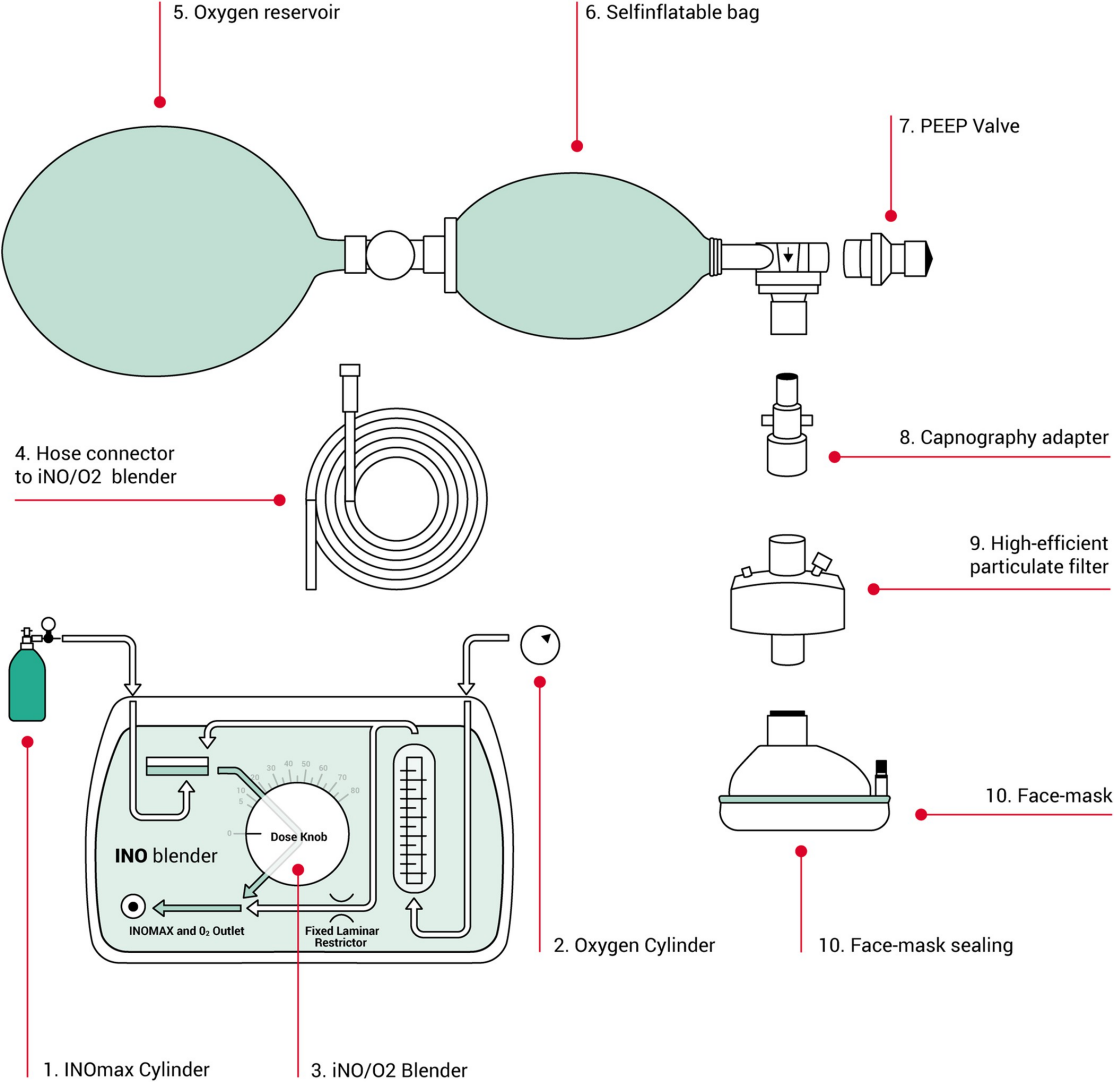
iNO equipment setup.

The main intervention of this trial is to add 20 ppm nitric oxide 800 ppm mol/mol (INOmax®) delivered by the INOBlender® to the medical oxygen delivered by bag/ mask to patients in acute respiratory failure (Fig 2 iNO equipment setup). All patients will receive three minutes with oxygen 10 l/min with PEEP valve set to 10cm H_2_O by sealed face mask prior to any screening for inclusion. If SpO_2_ remains <90% at this point, patient will be assessed and randomized to either adding iNO or continuing 100% oxygen only. If SpO_2_ is >90% after the initial three minutes, patients will be treated as per local guidelines outside study protocol.

Blinded treatment of 100% oxygen with or without 20 ppm of iNO will be administered for five minutes before any attempt to intubate (or decision not to) will be made.

RSI may be performed at the discretion of the study doctor in accordance with local guidelines, most commonly involving ketamine, fentanyl and rocuronium bromide. Following RSI or other reasons for the bag/mask system having been left idle, the ventilation bag is squeezed the 3–4 times with O_2_/iNO before re- starting ventilation. This is to empty the bag of any toxic NO_2_ that may have been built up. Regardless, whether RSI is performed, or bag/mask ventilation is continued, a dose reduction of iNO should be attempted if SpO_2_ is ≥94% after 5 minutes (t_1_) Reduction is made in steps of 5 ppm every 5 minutes unless SpO_2_ drops by > 5 percentage points from the value at t_1_. After arrival in hospital, when patient is intubated or a decision not to intubate has been made, iNO administered by INOBlender should be weaned by 5 ppm every 5 minutes. In the unlikely event of a rebound effect (as assessed by the study doctor) iNO may be continued temporarily as necessary. iNO administrated by other devices (i.e.: not IMP) may be initiated as per local routines at the discretion of the attending doctor taking over the responsibility for the patient.

Any additional medical intervention will be carried out as considered necessary by the attending doctor according to institutional guidelines and established clinical practice.

Expected side effects such as systemic hypotension, bradycardia, pulmonary hypotension, kidney failure and methaemoglobinaemia, and platelet will be monitored and recorded as secondary endpoints. The dosage of 20 ppm is in accordance with previous trials, case reports and clinical experience and found to be an adequate balance between sufficient effect and potential side effects [6,7,10–12,14].

### Comparisons and assessments

This trial compares the effect of adding iNO at 20 ppm to preoxygenation in patients with COVID-19 and respiratory failure. The primary endpoint is the change in oxygen saturation in the first five minutes after adding iNO. Despite a short-term primary endpoint, the study is designed with a 28-day follow up for assessment of mortality, SARS-CoV-2 status and a range of exploratory outcomes relating to iNO treatment and intensive care management. Please consult Fig 1, SPIRIT schedule.

#### Primary endpoint

Primary endpoint is assessed by measuring SpO_2_ non-invasively by pulse oximetry before (baseline (t_0_)) and five minutes after start of IMP administration (t_1_). SpO_2_ serves as an approximated measure of arterial oxygen saturation. C.f. Table 1. Study objectives and related endpoints.

**Table 1:** Study objectives and related endpoints.

	Objectives			Endpoints	Comment
	Primary			Primary	
1	Evaluate the clinical efficacy of iNO to increase oxygen saturation prior to, and during and after emergency RSI in patients with suspected or confirmed COVID-19 respiratory failure.		1.1.1	Δ SpO_2_ (t_1_-t_0_)	ΔSpO_2_: change in O_2_ saturation from t_0_ to t_1_
				Secondary	
			1.2.1	Δ SpO_2_ (t_2_-t_0_)	ΔSpO_2_: change in O_2_ saturation from t_0_ to t_2_
			1.2.2	SpO_2_ during RSI (t_RSI_)	Lowest measured value
			1.2.3	SpO_2_ from t_1_—t_n_	At 5 minutes intervals
			1.2.4	Number of patients with SpO_2_<50 from t_1_ to EOI	EOI: end of intervention
			1.2.5	Respiratory rate from t_0_ to t_RSI_ or EOI	Calculated from impedance
			1.2.6	PaO_2_ at t_0_ to EOI	Where available
			1.2.7	Cardiac arrest during intervention	Utstein definition
			1.2.8	GOS-E score on day 28	GOS-E: Glasgow Outcome Score Extended
			1.2.9	Mortality	24 hour and 28 days
	Secondary			Secondary	
2	Evaluate the safety of the intervention as compared to the control as assed by:	2.1 Circulatory function	2.1.1	Heart rate at t_0_-t_n_	Actual HR at time point
			2.1.2	Blood pressure at t_0_ to t_n_	Invasive or non-invasive. Actual BP at time point
		2.2 Kidney function	2.2.1	Increase in Serum urea concentration	Highest measured value up to day 28
			2.2.2	KDIGO AKI Stage up to day 28	Kidney Disease Improving Global Outcomes acute kidney injury stage
		2.3 Hemoglobin function	2.3.1	Arterial MetHgb concentration	Highest value during intervention or first sample after EOI
		2.4 Platelet count	2.4.1	Platelet count	Lowest value up to 7 days
			2.5.1	Need for iNO after EOI	Start time, total duration (days, hours), device and highest dose. If further iNO was continuation of intervention (y/n)
			2.5.2	ICD-10 diagnosis on discharge	
	Exploratory			Exploratory	
3.	Evaluate the clinical efficacy of iNO compared to standard treatment on respiratory severity and length of specialized care		3.1.1	O_2_-ratio for patients on mechanical ventilation first 24 hours	Lowest measured value. O_2_-ratio: PaO_2_/FiO_2_
			3.1.2	PaCO_2_ at t_0_ to EOI	
			3.1.3	EtCO_2_ at t_0_ to EOI	
			3.1.4	Ventilator free days up to day 28	
			3.1.5	Length of stay in ICU	
4	Evaluate the effect of iNO on cardiac stress		4.1.1	Serum troponin T	Highest measured level first 24 hours where available
			4.1.2	ProBNP	

#### Secondary endpoints

Secondary endpoints include change in SpO_2_ from baseline to 10 minutes after start of IMP administration (1.2.1) (1.2.4). Respiratory rate (1.2.5) reflects hypoxic drive and is expected to fall as SpO_2_ increases. Arterial blood gas analyses (1.2.6 and 1.2.7) are not easily obtainable in the pre-hospital environment but is considered a more accurate reflection of oxygen saturation. Where available, this measurement will be included in the analyses. On the extreme, cardiac arrest or severe bradycardia (1.2.8) are markers of hypoxemia with potentially lethal consequences and are included in the secondary analyses. Glasgow outcome score extended (GOS-E) may reflect brain damage following hypoxia (1.2.9). Mortality in 24 hours (1.2.9) may be direct result of hypoxemia in this patient group and a potential important marker for the efficacy of iNO. Mortality at 28 days is relevant as late complications following hypoxemia may manifest at this stage. Secondary endpoints also include known adverse effects of iNO. Although these are not described after short-term administration, they will be included in this study. Both bradycardia and hypotension are possible side effects of NO-gas. Heart rate and blood pressure are endpoints monitored every 5 minutes during the intervention phase. (2.1.1 and 2.1.2).

All other secondary outcome parameters are collected from the patient journal.

Acute kidney injury is classified in three stages according to Kidney Disease Improving Global Outcome Guidelines (KDIGO) [19]. (2.2.2) A more sensitive but less specific marker of kidney function is the change in serum urea concentration. Both measures are gathered on the 28-day follow-up, and the value most strongly indicative of kidney failure is used for this endpoint. Platelet count and methemoglobinemia are collected from the patient journals. Data from patient monitor measuring impedance (ohms) will be used to calculate respiratory rate. C.f. Table 1. Study objectives and related endpoints.

### Data collection, management and monitoring

Source data in this trial includes study specific paper case report form. In addition, all patient records may be sources of specific information. Data will be collected manually on study specific paper CRF at the inclusion visit (the acute event) and by day 28 visit. For patients in this trial this includes:

- Study specific paper CRF inclusion visit and day 28 visit
- Prehospital paper records, computer notes in LABAS, records in AMIS and sound or in medical dispatch system.
- Records from medical monitors such as LP15 and CorPulse
- In-hospital results in DIPS® or other EPJ

Further details on source data hierarchy are outlined in the EUDRACT protocol. Members of the steering group will access the OUH EPJ system to obtain the necessary information, and make sure that patients that are transferred or discharged from hospital are not lost to follow-up.

Data management will be performed by the data management unit at the Clinical trials unit (CTU), OUH [20]. The data management procedures will be performed in accordance with the department’s SOPs and ICH guidelines. The data management process will be described in the study specific data handling plan and the study specific data handling report after database closure. De-identified data will be stored in a dedicated and secured area. The data will be stored until 15-years following the last patient’s final study visit.

The clinical study monitor of the study is the monitoring unit at CTU, OUH [20]. The study coordinating group will be visited on a regular basis with a frequency found appropriate by the unit, to check the informed consent process, adherence to protocol, maintenance of required regulatory documents, data completion including source data verification, as well as safety monitoring as specified below.

### Discontinuation of interventions

Patients will not be able to discontinue treatment prior to end of intervention in the present trial but can discontinue after end of intervention but before the 28-day follow-up.

If SpO2 is < 50% at any point after allocation/randomization the patient case can be emergency unblinded and treatment continued as decided by study doctor. In case of cardiac arrest, iNO is continued at the discretion of the study doctor. In case a serious adverse reaction triggered by IMP is suspected, an emergency unblinding should be performed, and treatment with IMP discontinued or continued at the discretion of the study doctor.

### Safety monitoring and reporting

Definitions of adverse events follow the ICH GCP guidelines. The nature of the patient population studied, the pre-hospital environment, severity of illness included, and multitude of medical interventions makes the adverse events (AE) reporting challenging. Several events that meet the AE criteria set out in GCP are expected in the natural history of critical illness and in the treatment offered to patients included in this trial. On this basis we find support in the literature to prespecify outcomes that would be reported as AEs in other trials but are expected outcomes in the present [21,22]. The period for collecting and recording AE will be from allocation and until end of intervention, defined as the time point where iNO is discontinued or when care of the patients is transferred from pre-hospital doctor to the hospital. Serious adverse events will be reported to the study sponsor within 24 hours.

Post-trial care for included patients is equal to any patient not included in the study.

The principal investigator will obtain insurance coverage for this study through membership of the Drug Liability Association and ensure proof of membership is sent Competent Authority and stored in the trial master file prior to inclusion of the first patient.

### Trial discontinuation

The whole trial may be discontinued at the discretion of the principal investigator (PI) or the sponsor in the event of any of the following:

- Occurrence of AEs unknown to date in respect of their nature, severity and duration
- Medical or ethical reasons affecting the continued performance of the trial
- Difficulties in the recruitment of patients
- At sponsors discretion

The sponsor and principal investigator will inform all investigators, the relevant Competent Authorities and Ethics Committees of the termination of the trial along with the reasons for such action. If the study is terminated early on grounds of safety, the Competent Authorities and Ethics Committees will be informed within 15 days.

### Study coordinating group

Coordination of the study and training of personnel will be overseen by a steering group consisting of HJH (principal investigator), JH, MR, AS and FH. The steering group will ensure adequate training to ensure that recruiting clinicians have the proper knowledge to male correct inclusions/exclusions and skills to adhere to protocol. Heads of recruiting branches (HJH and AS) will monitor any occurrences of missed cases from the institution´s electronic patient journal system. Depending on the reasons for missing cases, measures such as additional individual training may be initiated by the steering committee. All investigators ascertain they will apply due diligence to avoid protocol deviations.

### Data sharing and publication

OUH has complete ownership of all data and publishing rights of all results.

The full protocol, Statistical Analysis Plan, information letter for consent and other trial documents will be published open access. The Clinical Study report and Statistical analysis report will also be made openly available, but may be altered to hide information that may lead to identification of individual study participants. These documents will be shared at Norwegian Centre for Research Data (NSD).

All of the individual participant data collected during the trial, after de- identification will be made available to anyone who wishes to access the data. Data will be made indefinitely available through Norwegian Centre for Research Data (NSD). De- identified data can only be distributed in accordance with the data processor agreement entered into between the Sponsor and NSD.

Data sharing with editors or peer- reviewers of scientific journals, conferences or the like will not require specific consent or data access agreement with Oslo University Hospital, in the understanding that the data will not be shared onward or used beyond reviewing this trial.

We aim to publish the results of this trial in an international peer reviewed journal with authorships based on the Vancouver Convention. Eligible authors are the authors of this protocol. We have no intention of using professional writers.

### Statistical analysis

#### Power calculation

One previous study in a different setting has shown that 71% of patients respond to the treatment with an absolute increase in SpO_2_ of >5 percentage points [23]. We assume a standard deviation of 10 percentage points, and a within subject correlation of 0.8 based on clinical experience. This implies a standard deviation of the change in SpO_2_ (after minus before intervention) of 6.32%. A difference in improvement in SpO_2_ of 5 percentage points between the intervention groups is considered clinically significant. To identify a difference of improvement of SpO_2_ of 5 percentage points, with a power of 80%, we need to include 54 patients (27 in each arm) in this trial, using a significance level of 5%.

Because of the uncertainty related in the magnitude of the variance in the SpO_2_ change, a simple blind re-estimation of the sample size based on the variance from a subsample of the 20 first patients will be done. The sample size will be adjusted if necessary, but the total sample will not be reduced (to less than 54). By using a blinded one-sample variance estimator for sample size recalculation, the effect of sample size adjusting on the type I error rate is negligible and no additional measures are needed to control the significance level [24].

#### Primary endpoint

The primary efficacy endpoint is the difference in the change in SpO_2_ from before to after the intervention. The null hypothesis is that there is no difference in improved oxygen saturation between administering oxygen/iNO mixture to spontaneously breathing patients with COVID-19 related hypoxia, compared to oxygen alone. The alternative hypothesis is that there is a difference. The primary endpoint will be analyzed using a linear regression model where the intervention variable will be adjusted by the baseline SpO_2_. If more than one center is included, and used as a stratification variable in the randomization, this will also be adjusted for in the model. There will be one safety interim analysis in this trial, and one main efficacy analysis at the end of the trial.

### Trial status

Given the current pandemic development in our region, no patients have been recruited to this trial. Any significant protocol modifications prior to, or during the study will undergo formal application to national medical agency and published through the WHO/EUDRACT system.

### Declarations

#### Ethics approval and consent to participate

REK-128904 (c.f. Additional file II in S1 Study protocol). The INOCOV study has not yet recruited any patients.

#### Trial sponsor

Christian Buskop, MD, Head of Clinic, Air Ambulance Department, Division of Prehospital Services, Postbox 4956 Nydalen; 0424 Oslo, Norway Phone: +47 99438374, E-mail: cbuskop@ous-hf.no.

#### Consent for publication

All authors read and approved the protocol manuscript.

#### Trial status

The INOCOV study has not yet recruited any patients.

## Supporting information

S1 Checklist SPIRIT checklist. (DOC)

S1 Study protocol Ethical approval (translated version). (PDF)

## Abbreviations

AE: adverse events
AKI: Acute kidney injury
COVID-19: Corona virus disease
iNO: Inhaled nitric oxide
CA: Cardiac arrest
CPAP: Continuous positive airway pressure
EPJ: Electronic patient journal
EOI: End of intervention
E/D: Early discontinuation
ppm: parts per million
RSI: Rapid sequence induction
SPIRIT: Standard protocol items for clinical trials
ICH: International conference on harmonization of technical requirements for registration of pharmaceuticals for human use
ICU: Intensive care unit
IMP: Investigational medical product
OUH: Oslo university hospital
PI: Principal investigator
HEMS: Helicopter emergency medical service
PEEP: Positive end-expiratory pressure
GOS-E: Glasgow outcome score extended
NSD: Norwegian centre for research da
SAE: Serious adverse events
SAP: Statistical analysis plan
