# Supplementary material for: Inhaled nitric oxide as temporary respiratory stabilization in patients with COVID-19 related respiratory failure (INOCOV): Study protocol for a randomized controlled trial
Source: PLoS One. 2022 May 27;17(5):e0268822. doi: 10.1371/journal.pone.0268822 (PMC9140246; doi:10.1371/journal.pone.0268822)
Supplement: S1 Study protocol — (PDF) [file pone.0268822.s002.pdf]

[redacted]

INHALED NITRIC OXIDE AS A BRIDGE TO MECHANICAL VENTILATION IN PATIENTS WITH SUSPECTED COVID-19 RESPIRATORY FAILURE.

A RANDOMIZED, BLINDED TRIAL COMPARING INHALED NITRIC OXIDE ADDED TO PREOXYGENATION STANDARD PROCEDURE PRIOR TO RAPID SEQUENCE INTUBATION

Protocol Identification Number: INOCOV-19
EudraCT Number: 2020-001656-18
WHO Universal Trial Number: U1111-1250-1698

SPONSOR:	Oslo University Hospital<br>Christian Buskop, MD, Head of Department<br>Air Ambulance Department<br>Division of Prehospital Services
PRINCIPAL INVESTIGATOR (PI):	Hans Julius Heimdal, MD<br>Address: Sykehusveien 19,<br>1474 Nordbyhagen, Norway<br>Tel : + 47 95251053<br>E-mail: hanhei@ous-hf.no

PROTOCOL VERSION NO. 1.1 - 29.04.2020

CONTACT DETAILS

**Sponsor:**	Christian Buskop, MD, Head of Department<br>Air Ambulance Department<br>Division of Prehospital Services<br>Postbox 4956 Nydalen; 0424 Oslo, Norway<br>Phone: +47 99438374<br>E-mail: cbuskop@ous-hf.no
**Principal investigator**	Hans Julius Heimdal, MD<br>Luftambulansebasen<br>Address: Sykehusveien 19,<br>1474 Nordbyhagen, Norway<br>Phone : + 47 95251053<br>E-mail: hanhei@ous-hf.no
**Participating Departments:**	Oslo University Hospital<br>Postboks 4956 Nydalen;<br>0424 Oslo, Norway
**Monitor:**	Oslo universitetssykehus HF<br>Avdeling Forskningsstøtte for kliniske studier - Clinical Trial Unit (CTU)<br>Postboks 4950 Nydalen, 0424 Oslo<br>Tel: (+47) 915 02770<br>E-mail: oushfpbctu@ous-hf.no
**Pharmacy:**	Hospital pharmacy South-Eastern Norway Regional Health Authority (Oslo University Hospital)<br>Stenersgt. 1A, postkasse 79, 0050 Oslo<br>Telephone (+47) 23 13 52 00<br>Email: post@sykehusapotekene.no

SIGNATURE PAGE

**Title**	INHALED NITRIC OXIDE AS A BRIDGE TO MECHANICAL VENTILATION IN PATIENTS WITH SUSPECTED COVID-19 RESPIRATORY FAILURE.
**Protocol ID no:**	INOCOV-19
**EudraCT no:**	2020-001656-18
**Version and date**	PROTOCOL VERSION NO 1.1 Date 29.04.2020

I hereby declare that I will conduct the study in compliance with the Protocol, ICH GCP and the applicable regulatory requirements:

Name	Title	Role	Signature	Date
Christian Buskop	Consultant Anesthesiologist	Sponsor		
Hans Julius Heimdal	Consultant Anesthesiologist	PI		
Terje Strand	Consultant Anesthesiologist	Investigator		
Marius Rehn	PhD, Consultant Anaesthesiologist	Investigator		
Jostein Hagemo	PhD, Consultant Anaesthesiologist	Investigator		
Fridtjof Heyerdahl	PhD, Consultant Anaesthesiologist	Investigator		
Arne Kristian Skulberg	PhD, Consultant Anaesthesiologist	Investigator		
Morten Valberg	PhD	Statistician		

TABLE OF CONTENTS

CONTACT DETAILS **2**
SIGNATURE PAGE **3**
TABLE OF CONTENTS **4**
LIST OF ABBREVIATIONS AND DEFINITIONS OF TERMS **8**
1 INTRODUCTION **9**
1.1 Background 9
1.1.1 WHO Case definitions for surveillance of covid-19: 9
1.1.2 Clinical Aspects and Pre-hospital Considerations 9
1.2 Background - Therapeutic Information and clinical experience 11
1.2.1 Standard prehospital treatment in respiratory failure 11
1.2.2 Mechanism of action of inhaled nitric oxide 12
1.3 Rationale for the Study and Purpose 13
2 STUDY OBJECTIVES AND RELATED ENDPOINTS **14**
3 OVERALL STUDY DESIGN **16**
4 STUDY POPULATION **17**
4.1 Selection of Study Population 17
4.2 Number of Patients 17
4.3 Inclusion Criteria 17
4.4 Exclusion Criteria 17
5 TREATMENT **18**
5.1 Investigational Medicinal Product (IMP) 18
5.2 Drug Identity, Supply and Storage 18
5.3 Dosage and Drug Administration 18
5.3.1 INO delivery system and study setup 19
5.4 Premedication and Monitoring 20
5.4.1 Premedication: 20
5.4.2 Monitoring and equipment 20
5.5 Concomitant Medication 21
5.6 Drug Accountability 22
5.7 Drug Labelling 22
5.8 Subject Numbering 22
6 STUDY PROCEDURES **23**

6.1.1 Before Treatment Starts 23
6.1.2 Treatment 23
6.1.3 End of Intervention 23
6.1.4 Follow up visit 23
6.1.5 End of Trial 24
6.1.6 Flow Chart 25
6.1.7 Schedule of Activities 26
6.2 Criteria for Patient Discontinuation 28
6.3 Trial Discontinuation 28
6.4 Laboratory Tests 28
7 ASSESSMENTS 28
7.1 Assessment of Efficacy 28
7.1.1 Primary endpoint: Oxygen saturation 28
7.1.2 Secondary endpoints: 29
7.2 Safety Considerations and Assessments 30
7.3 Risk/ benefit balance of the trial 31
8 SAFETY MONITORING AND REPORTING 33
8.1 Definitions 33
8.1.1 Adverse Event (AE) 33
8.1.2 Serious Adverse Event (SAE) 33
8.1.3 Adverse Reaction (AR): 33
8.1.4 Suspected Unexpected Serious Adverse Reaction (SUSAR) 33
8.2 Expected Adverse Events 34
8.2.1 Expected Adverse Events from Inhaled NO 34
8.2.2 Other Expected Serious Adverse Events in current trial 34
8.3 Time Period for Reporting AE and SAE 36
8.4 Recording of Adverse Events 36
8.5 Reporting Procedure 37
8.5.1 SUSARs 38
8.5.2 Annual Safety Report 39
8.5.3 Clinical Study Report 39
8.6 Procedures in Case of Emergency and unblinding 39
8.7 Data Monitoring Committee (DMC) 39
9 DATA MANAGEMENT AND MONITORING 40
9.1 Case Report Forms (CRFs) 41

9.2 Source Data ... 41
9.3 Study Monitoring ... 43
9.4 Confidentiality ... 43
10 STATISTICAL METHODS AND DATA ANALYSIS ... 44
10.1 Determination of Sample Size ... 44
10.2 Randomization and Blinding ... 44
10.3 Population for Analysis ... 45
10.4 Planned analyses ... 45
10.5 Statistical Analysis ... 46
11 STUDY MANAGEMENT ... 46
11.1 Trial Master File (TMF) ... 46
11.2 Investigator Delegation Procedure ... 46
11.3 Protocol Adherence ... 47
11.4 Study Amendments ... 47
11.5 Audit and Inspections ... 47
12 ETHICAL AND REGULATORY REQUIREMENTS ... 47
12.1 Ethics Committee Approval ... 47
12.2 Other Regulatory Approvals ... 47
12.3 Informed Consent Procedure ... 47
12.4 Subject Identification ... 48
13 TRIAL SPONSORSHIP, OWNERSHIP AND FINANCING ... 48
14 TRIAL INSURANCE ... 49
15 PUBLICATION POLICY ... 49
16 DATA SHARING ... 49
17 LIST OF APPENDICES ... 50
18 REFERENCES ... 50

Protocol version and date	Amendment/ Change	Approved Ethics Committee	Approved Medicines Agency
Protocol versions with all changes marked in yellow between versions are kept in the Trial Master File			
v. 1.0 22.04.2020	- Original protocol submission	Not submitted	Not approved
v. 1.1 29.04.2020	Changes made relating to:<br>• Description of unblinding<br>• Pre-specification of primary end-point analysis and stratification<br>• Specifications regarding statistical populations between ITT and FAS<br>• Specifications regarding final Covid-19 diagnosis as statistical sub-group<br>• Confirmation that all women of less than 50 years of age will be asked about possible pregnancy<br>• stop-criteria for the IMP administration is updated.<br>• Medical oxygen has been defined as non-IMP in updated protocol<br>• Statement regarding the use of IMP within MA is updated<br>• Specification to exclusion criterion number 8		

LIST OF ABBREVIATIONS AND DEFINITIONS OF TERMS

Abbreviation or special term	Explanation
AE	Adverse Event
AMIS	Computer registry at AMK
AMK	Emergency Medical Dispatch Centre
ARDS	Acute respiratory distress syndrome
COVID-19	Disease caused by SARS-CoV-2 coronavirus
CRF	Case Report Form (electronic/paper)
CSA	Clinical Study Agreement
CTC	Common Toxicity Criteria
CTCAE	Common Terminology Criteria for Adverse Event
DAE	Discontinuation due to Adverse Event
DMC	Data monitoring committee
EC	Ethics Committee, synonymous to Institutional Review Board (IRB) and Independent Ethics Committee (IEC)
ECG	Electrocardiogram
EOI	End of intervention
EPJ	Electronic Patient Journal
$EtCO_2$	The amount of carbon dioxide (CO_2) in exhaled air
GCP	Good Clinical Practice
IB	Investigator's Brochure
ICF	Informed Consent Form
ICH	International Conference on Harmonization
IMP	Investigational medicinal product
INO	Inhaled Nitric Oxide
KDIGO	Kidney Disease Improving Global Outcomes (KDIGO)
LABAS	Electronic patient journal in OUH Air Ambulance Service
Norcrin	Norwegian Clinical Research Infrastructure Network
OUH	Oslo University Hospital
OUH	Oslo University Hospital
PEEP	Positive end-expiratory pressure
ppm	Parts per million
RSI	Rapid Sequence Induction
SAE	Serious Adverse Event
SARI	Severe acute respiratory infection
SARS-CoV-2	Novel coronavirus causing covid-19 disease
SOP	Standard Operating Procedure
SPC	Summary of product characteristics
SpO_2	Oxygen saturation
SUSAR	Suspected Unexpected serious adverse reaction
TMF	Trial master file
V/Q ratio	V – ventilation – the air that reaches the alveoli<br>Q – perfusion – the blood that reaches the alveoli via the capillaries<br>The V/Q ratio can therefore be defined as the ratio of the amount of air reaching the alveoli per minute to the amount of blood reaching the alveoli per minute
WHO	The World Health Organization

1 INTRODUCTION

1.1 Background

SARS-CoV-2 and COVID-19 epidemiology
In December 2019, several unexplained viral pneumonia cases occurred in Wuhan, China. Etiological research conducted identified what was later to be named the severe acute respiratory syndrome coronavirus 2 (SARS-CoV-2). The World Health Organization formally named the disease triggered by SARS-CoV-2 the coronavirus disease (COVID-19) (1). In March 2020, WHO announced the COVID-19 a pandemic and a major global public health emergency(2). Mortality from COVID-19 is rapidly increasing in Europe and the USA, with acute respiratory failure as the predominant cause of death(3).

1.1.1 WHO Case definitions for surveillance of covid-19:

Suspected case
A. A patient with acute respiratory illness (fever and at least one sign/symptom of respiratory disease, e.g., cough, shortness of breath), AND a history of travel to or residence in a location reporting community transmission of COVID-19 disease during the 14 days prior to symptom onset;
OR
B. A patient with any acute respiratory illness AND having been in contact with a confirmed or probable COVID-19 case (see definition of contact) in the last 14 days prior to symptom onset;
OR
C. A patient with severe acute respiratory illness (fever and at least one sign/symptom of respiratory disease, e.g., cough, shortness of breath; AND requiring hospitalization) AND in the absence of an alternative diagnosis that fully explains the clinical presentation.

Probable case
A. A suspect case for whom testing for the COVID-19 virus is inconclusive.
OR
B. A suspect case for whom testing could not be performed for any reason.

Confirmed case
A person with laboratory confirmation of COVID-19 infection, irrespective of clinical signs and symptoms.

1.1.2 Clinical Aspects and Pre-hospital Considerations

COVID-19 is characterized by an initial phase of unspecific symptoms. Due to risk of disease transmission, patients are generally advised to stay at home, unless medical care is needed. A significant proportion of these cases develop critical respiratory failure so rapidly that initial emergency medical management is required in the pre-hospital phase.

Already during the initial phase of the outbreak in Norway, Oslo University Air Ambulance Department encountered patients with life threatening respiratory failure, resembling a clinical picture similar to acute respiratory distress syndrome (ARDS), with severe hypoxia. The WHO also reports similar clinical picture in covid-19 patients, with rapidly progressive life- threatening respiratory failure. The WHO recommendations laid

out 13th March 2020 form the basis of the present trial protocol and is found as an appendix.

- **Management of severe COVID-19: oxygen therapy and monitoring:**
  Give supplemental oxygen therapy immediately to patients with SARI and respiratory distress, hypoxaemia or shock, and target $SpO_2 > 94\%$.
  Remarks for adults: Adults with emergency signs (obstructed or absent breathing, severe respiratory distress, central cyanosis, shock, coma or convulsions) should receive airway management and oxygen therapy during resuscitation to target $SpO_2 \geq 94\%$. Initiate oxygen therapy at 5 L/min and titrate flow rates to reach target $SpO_2 \geq 93\%$ during resuscitation; or use face mask with reservoir bag (at 10–15 L/min) if patient in critical condition

- **Management of critical COVID-19: ARDS**

Recognize severe hypoxemic respiratory failure when a patient with respiratory distress is failing to respond to standard oxygen therapy and prepare to provide advanced oxygen/ventilatory support.
Remarks: Patients may continue to have increased work of breathing or hypoxemia even when oxygen is delivered via a face mask with reservoir bag (flow rates of 10–15 L/min, which is typically the minimum flow required to maintain bag inflation; FiO_2 0.60–0.95). Hypoxemic respiratory failure in ARDS commonly results from intrapulmonary ventilation-perfusion mismatch or shunt and usually requires mechanical ventilation.
Endotracheal intubation should be performed by a trained and experienced provider using airborne precautions.
Remarks: Patients with ARDS, especially young children or those who are obese or pregnant, may desaturate quickly during intubation. Pre-oxygenate with 100% FiO_2 for 5 minutes, via a face mask with reservoir bag, bag-valve mask, HFNO or NIV. Rapid-sequence intubation is appropriate after an airway assessment that identifies no signs of difficult intubation.

As stated above, patients in severe respiratory failure will require mechanical ventilation. Mechanical ventilation is artificial ventilation where spontaneous breathing is replaced with a machine (ventilator/ respirator/ reinflatable bag) breathing into the patients' lung via an endotracheal tube. The placing of an endotracheal tube in emergency settings, as opposed to elective, is referred to as Rapid Sequence Intubation (RSI). To achieve intubation and mechanical ventilation the patient needs to be anesthetized.
In order to perform an RSI and endotracheal intubation, the patients need preoxygenation. The procedure itself is a high-risk medical intervention, particularly when the indication for RSI is expiratory failure and hypoxaemia.
To increase the safety margins preoxygenation by applying a closed facemask with high flow oxygen and positive end-expiratory pressure (PEEP). Intubation follows standard guidelines in the service that describe equipment and drugs used for the procedure.

1.2 Background - Therapeutic Information and clinical experience

1.2.1 Standard prehospital treatment in respiratory failure

Respiratory failure is a common presentation in emergency medicine, and its treatment is both aimed at identifying and treating the cause of the problem, and immediate symptomatic treatment. Intervention follows the basic algorithm common to all emergency medicine: A, B, C; meaning Airway, Breathing, Circulation; with interventions falling under each category and the order for these interventions going from A through to C.

Airway: this means securing the patients has a free and unobstructed airway from the mouth and down though the pharynx and trachea into the lungs. Interventions under A include removing foreign objects, chin- and jaw lift to avoid the base of the tongue blocking the airway, insertion of a Guedel- airway or endotracheal intubation. The endotracheal intubation is considered a “secure- airway” and is often part of physician-manned emergency care and prior to transport. There may me instances where the intubation is delayed until arrival at the hospital and the treating emergency doctor chooses to bag- mask ventilate the patient outside of the hospital and during transport.

Breathing: Interventions under B includes the administration of oxygen, either via face masks and nasal prongs in patients with spontaneous respiration, or via bag- mask ventilation of more invasive airway devices such as intubation. Mechanical ventilation is included under this point.

Circulation: Intervention under this point includes the administrating of IV fluids, transfusion, medication for blood pressure such as adrenalin, noradrenalin or others.

For endotracheal intubation most patients, require deep sedation and muscle relaxation- the induction of anaesthesia. Common dugs to obtain this is ketamine or propofol for sedation and suxamethonium or rocuronium bromide for relaxation. These drugs may be administered IV or interosseous, rarely intramuscularly. For the safest possible intubation, a period of preoxygenation must occur. This involves saturating the patient's lungs and blood stream with oxygen, and the intubation invariably induces a short time period where the patient is apnoeic. During this time patients may have a considerable drop in their SpO_2, especially if they are critically ill. This is a central rationale for the present trial, to increase the oxygen uptake during this preoxygenation phase.

1.2.2 Mechanism of action of inhaled nitric oxide

Inhaled nitric oxygen (INO) causes local pulmonary vasodilation by rapidly diffusing across alveolar cells to the neighbouring smooth muscle of pulmonary arterioles, where it activates cyclic GMP (see illustration below) (4). Nitric oxide (NO) is an important determinant of local blood flow and is formed by the action of NO synthase (NOS) on the semi-essential amino acid L-arginine in the presence of molecular oxygen. INO results in preferential pulmonary vasodilatation and lowers pulmonary vascular resistance and improves oxygenation(5).

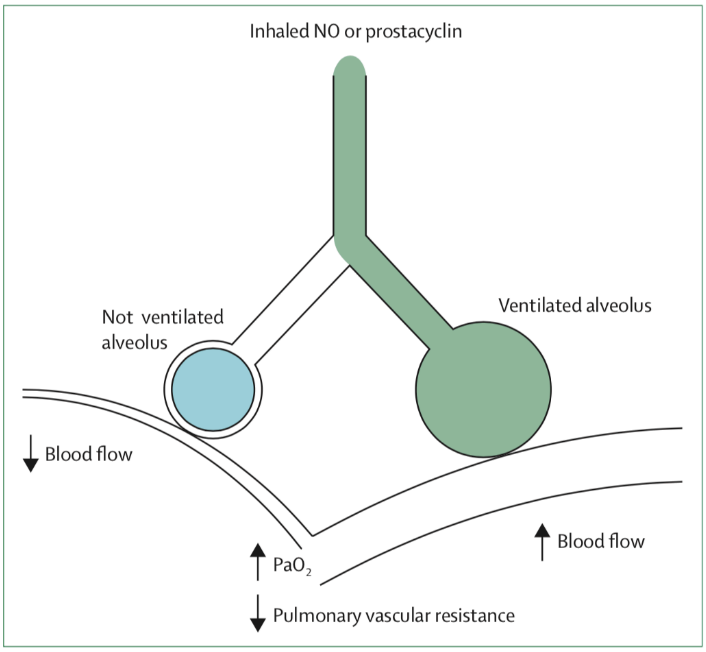

***Figure 3:* Selective pulmonary vasodilation**
Inhaled pulmonary vasodilators like nitric oxide (NO) and prostacyclin are selectively delivered to the part of the lung that is ventilated. The result is an improvement in arterial oxygen partial pressure (PaO_2) and a decrease in pulmonary vascular resistance. ↑=increase. ↓=decrease.

Clinical Use of Inhaled Nitric Oxide

INO has been in clinical use since the early 1990s(6). It is a highly reactive gas with an in vivo half-life of three to five seconds. Consequently, continuous inhalation is required to obtain clinical effect. One major benefit from the inhaled route of administration is selective vasodilation of well-ventilated pulmonary vessels. This reduces ventilation-perfusion mismatch, which is one of the key features of ARDS. Some adverse effects have been reported after long-term use, of which kidney failure is the most important. Impairment of surfactant function, alterations of immune system and platelet function, as well as circulatory effects has also been reported, but the clinical relevance remains uncertain.

Several studies find that INO rapidly increases the ratio of oxygen partial pressure in the blood to inspired oxygen concentration (PaO_2/FiO_2 ratio)(6). This effect appears to be transient, lasting less than 72 hours. A recent systematic review concludes that there is currently insufficient evidence to support long-term INO in hypoxic respiratory failure. Nevertheless, INO used as a temporary measure on certain indications is widespread in both neonatal and adult critical care(7). Except for neonatal critical care, INO is most frequently used as a rescue therapy in adult patients with hypoxic respiratory failure, or as a bridge to extracorporeal membrane oxygenation(8).

In close co-operation with intensive care units in Oslo University Hospital (OUH), the INO option has been implemented in the Air Ambulance Department since 2002, and nearly 200 treatments during transport has been documented, 71% of patients are reported to respond to the treatment in this setting (internal data OUH). A case report of local practice has been published(9).

A Cochrane meta-analysis found no statistically significant effects of INO on long term mortality in patients with ARDS. However, the use of INO gave significant improvements in oxygenation in the short term, measured at 4 hours in the review.(7)

In terms of safety, Cochrane reports a statistically significant increase in renal failure in the INO groups. Interestingly this adverse event is not noted in the SPC chapter 4.8 Undesirable effects. The Cochrane analysis includes studies on this adverse event in patients admitted to hospital and intensive care units. A systematic review from 2015 finds a dose- response relationship between INO and renal failure. A reduced dose and length of INO treatment reduces the risk of kidney failure(10)

In Scandinavian guidelines for the treatment of ARDS they advise against routine use of INO, but recognize that INO may be used as a rescue measure to temporarily increase oxygenation in patients with catastrophic hypoxemia and imminent risk of death (weak recommendation, moderate quality of evidence)(11). The literature has described INO being used as rescue strategy in severe ARDS attributable to 2009 H1N1 influenza(12).
For prehospital use of INO as emergency treatment to improve oxygenation prior to intubation there are no literature available. A PubMed central search 31. March 2020 yields no results for: "Rapid Sequence Induction and Intubation"[Mesh]) AND "Nitric Oxide"[Mesh].
Recent experience during the COVID-19 pandemic indicates a need for additional measures to rapidly improve oxygen saturation for safe transport or rapid sequence induction, intubation and mechanical ventilation. As a direct consequence of this situation, a standard operating procedure for pre-hospital INO as a rescue therapy for patients with COVID-19, has been implemented at Oslo University Hospital. We also want to explore this option in a trial setting, as the evidence is inconclusive.

1.3 Rationale for the Study and Purpose

The rationale for the present trial is to make airway management and intubation safer in patients with confirmed or suspected covid-19 and who suffer from acute respiratory failure and/or severe hypoxaemia.
A randomized controlled trial with blinding is well recognized as the most appropriate design to evaluate new medical interventions.
Choice of inhaled nitric oxide
Patients suffering from Covid-19 and with severe respiratory failure require rapid intubation for two reasons. Firstly, intubation is required to treat their acute respiratory distress syndrome and secure oxygenation. Secondly, endotracheal intubation with a closed circuit and expiratory filters reduces the spread of droplets and reduces exposure of health personnel and others to SARS-CoV-2 virus particles.
Emergency intubation without sufficient preoxygenation is a high- risk medical procedure. It holds significant risk for the patient, with oxygen desaturation potentially leading to hypoxic brain injury, cardiac arrest or death. In covid-19 and other acute respiratory failure situations, hypoxemic respiratory failure commonly results from intrapulmonary ventilation-perfusion mismatch ((V/Q ratios) or shunt. This is the precise mechanism of action and physiological mechanism in which inhaled nitric oxide works. Quoting from the SPC: “When inhaled, nitric oxide produces selective pulmonary vasodilation. INOmax (the drug under study) appears to increase the partial pressure of arterial oxygen (PaO_2) by dilating pulmonary vessels in better ventilated areas of the lung, redistributing pulmonary blood flow away from lung regions with low ventilation/perfusion (V/Q) ratios toward regions with normal ratios.”
Choice of dose and way of administration
We have chosen the common and approved starting dose of inhaled nitric oxide (INO); 20 ppm. In out trial there is no titration upwards. The rationale for this is an assumption that effect will be seen at this starting dose. Titration of dose is not feasible within the short time span of treatment in the present trial.
INO is approved for endotracheopulmonary use, meaning administration after intubation through an endotracheal tube. However, delivery systems for administration through manual resuscitator bag or nasal cannulas are also approved. In the current trial we will administer the drug using the INOblender® (INO Therapeutics LLC, Mallinckrodt Manufacturing LLC, Madison, Wisconsin, USA).
The device is well suited for our trial as it is approved for short term attended use when a primary delivery device cannot practicably be used. This intended use includes applications within a medical facility and transport outside of a medical facility.
There are no alterations to the setup of medical devices in this trial, and in line with the relevant CE approvals.

2 STUDY OBJECTIVES AND RELATED ENDPOINTS

The overall objective of the study is to evaluate the clinical efficacy and safety of INO compared to standard treatment prior to and during emergency RSI in hypoxic patients with suspected or confirmed COVID-19. The primary and secondary and exploratory objectives of this study is listed below, with associated endpoints.

	Objectives			Endpoints	Comment
	Primary			Primary	
1	Evaluate the clinical efficacy of INO to increase oxygen saturation prior to, and during and after emergency RSI in patients with suspected or confirmed COVID-19 respiratory failure.		1.1.1	Δ SpO_2 (t_0-t_1)	ΔSpO_2: change in O_2 saturation from t_0 to t_1
				Secondary	
			1.2.1	Δ SpO_2 (t_0-t_2)	ΔSpO_2: change in O_2 saturation from t_0 to t_2
			1.2.2	SpO_2 during RSI (t_{RSI})	Lowest measured value
			1.2.3	SpO_2 from t_1 - t_n	At 5 minutes intervals
			1.2.4	Number of patients with SpO_2<50 from t_1 to EOI	EOI: end of intervention
			1.2.5	Respiratory rate from t_0 to t_{RSI} or EOI	Calculated from impedance (ohms).
			1.2.6	PaO_2 at t_0 to EOI	Where available
			1.2.7	Cardiac arrest during intervention	Utstein definition (13)
			1.2.8	GOS-E score on day 28	GOS-E: Glasgow Outcome Score Extended
			1.2.9	Mortality	24 hour and 28 days
	Secondary			Secondary	
2	Evaluate the safety of the intervention as compared to the control as assed by:	2.1 Circulatory function	2.1.1	Heart rate at t_0-t_n	Actual HR at time point
			2.1.2	Blood pressure at t_0 to t_n	Invasive or non-invasive. Actual BP at time point
		2.2 Kidney function	2.2.1	Increase in Serum urea concentration	Highest measured value up to day 28
			2.2.2	KDIGO AKI Stage up to day 28	Kidney Disease Improving Global Outcomes acute kidney injury stage (1-3)
		2.3 Hemoglobin function	2.3.1	Arterial MetHgb concentration	Highest value during intervention or first sample after EOI
		2.4 Platelet count	2.4.1	Platelet count	Lowest value up to 7 days

		2.5.1	Need for iNO after EOI	Start time, total duration (days, hours), device and highest dose. If further INO was continuation of intervention (y/n)
		2.5.2	ICD-10 diagnosis on discharge	
	Exploratory		Exploratory	
3.	Evaluate the clinical efficacy of INO compared to standard treatment on respiratory severity and length of specialized care	3.1.1	O_2-ratio for patients on mechanical ventilation first 24 hours	Lowest measured value. O_2-ratio: PaO_2/FiO_2
		3.1.2	$PaCO_2$ at t_0 to EOI	
		3.1.3	$EtCO_2$ at t_0 to EOI	
		3.1.4	Ventilator free days up to day 28	
		3.1.5	Length of stay in ICU	
4	Evaluate the effect of INO on cardiac stress	4.1.1	Serum troponin T	Highest measured level first 24 hours where available
		4.1.2	ProBNP	

t_0 : time of allocation

t_1 : 5 minutes after initiation of iNO

t_2 : 10 minutes after initiation of iNO

3 OVERALL STUDY DESIGN

The study is a phase II drug trial of inhaled nitric oxide as an adjunct to standard of care with medical oxygen as part of airway management in patients with known or suspected covid-19 and who are in acute respiratory failure.

The trial is parallel two-arm, randomized, controlled blinded trial.

The primary outcome measure is the change in SpO_2, and the null hypothesis is that there is no difference in the change in SpO_2 following initiation of INO.

The trial is initiated as a single center study but is open for inclusion from other centers that have INO delivered by INOBlender as a treatment option.

**Study Period**	Estimated date of first patient enrolled: 01. May 2020<br>Anticipated recruitment period: 24 months<br>Estimated date of last patient completed: 30. April 2021
**Intervention Duration:**	Estimated one hour but will differ significantly due to the prehospital environment. From inclusion to handing over care time will depend on evacuation and transport times.
**Follow-up:**	After End of Intervention, participants will be followed up at day 28. This follow up visit will be a chart- review with or without interview with treating physician at main hospital admission from end of intervention till day 28. For patients still admitted at day 28 and discharged prior to end of trial their discharge note will be assessed.

4 STUDY POPULATION

4.1 Selection of Study Population

Study setting
The study will be conducted at Oslo University Hospital. The Prehospital division is the center. This center consists of two physician manned helicopters (coded 1-1 and 1-2 or 1-7) based in Lørenskog, Viken, Norway, one physician manned ambulance in Oslo (coded 119) and a search and rescue helicopter (coded 1-8) based in Rygge, Viken; Norway.
Other sites treating emergency cases in Oslo University Hospital such as intensive care units and emergency departments at Rikshospitalet and Ullevål may be included without substantial amendments to the present protocol.
In the case of expansion to sites outside of Oslo University Hospital amendment to the protocol will be made and competent authorities notified.

4.2 Number of Patients

Minimum 54 patients will be included in this trial.

4.3 Inclusion Criteria

All criteria must apply

1. Patients ≥ 18 years of age at the time of inclusion
   and
2. who are confirmed, suspected or probable cases of covid-19 based on WHO definitions
   and
3. SpO_2 <90% after 3 minutes 10 cm H_2O PEEP + 100% O_2
   and
4. who are treated by a physician who have specifically undergone formal training in pre-hospital INO administration and the present protocol.

4.4 Exclusion Criteria

Only one criterion needs to apply for exclusion:

1. Patients who after 3 mins of ventilation with 10 cm H_2O PEEP + 100% O_2 i.e. at time of allocation (t_0), have a SpO_2 <50%, or a reliable SpO_2 is impossible to obtain
2. Patients in severe kidney failure or dialysis,
3. Known or suspected pregnancy based on information on the time of inclusion.
4. Hypersensitivity to the active substance (NO) or to the excipient (N2)
5. Cardiac arrest
6. Patient in prison or custody by police
7. Staff present in treatment situation known to be pregnant
8. Any reason why, in the opinion of the treating physician, it is probably in the best interest of the patient not to participate in the trial.

Female participants under the age of 50 years will be asked explicitly about possible pregnancy. If this cannot be obtained from the patient, next of kin will be asked if present. Information regarding exclusion criteria 2, 3 and 4 will be obtained from bystanders, often relatives or friends present at time of inclusion. If no clear information is available, the physicians shall include or exclude based on his/her best judgment at the time of inclusion.

5 TREATMENT

5.1 Investigational Medicinal Product (IMP)

For this study the IMP is:

INOmax 800 ppm mol/mol delivered by the INOBlender, is defined as the Investigational Medicinal Product (IMP). The study set up is depicted in 5.3.1.
The INOMax + INOblender set up described is added onto the standard set up in the service for the administration of medical oxygen on bag-mask system. There are no alterations to either drug or drug delivery system in this trial outside of approvals given for the equipment and its use.

5.2 Drug Identity, Supply and Storage

The drug studied, inhaled nitric oxide, will be delivered by Linde Healthcare (Linde Gas AB – Linde Healthcare, Rättarvägen 3, 169 68 Solna, Sweden) as INOmax 800 ppm 2 Litre gas cylinders. They will be stored at Lørenskog Helicopter base in accordance with the specifications laid out in the SPC. The INOmax gas cylinders for this trial will be stored separate from the gas cylinders used in other clinical practice.
All documentation relating to the order, delivery and receiving of INOmax 800 ppm mol/mol for this trial will be kept in the TMF with a drug accountability system being in place for the identification of batch numbers of gas-cylinders and which INOBlender was used at each patient.

5.3 Dosage and Drug Administration

In this trial we follow the SPC of INOMax and start dosing at 20 ppm. We will not allow doses above this within this protocol. Weaning will follow standard practice. The duration of treatment is significantly lower than with the SPC, due to the objective of the trial- increased oxygen saturation prior to RSI

After 3 minutes on standard treatment (O_2 12l/min + 10 cm H_2O PEEP) patients with a SpO_2 between 50 and 90% will be randomized. For patients randomized to O_2 + Inhaled nitic oxide the drug will be administered in the following way:

- INOmax 800 ppm mol/mol will be administered by the INOBlender (IMP) at 20 ppm by bag-mask ventilation. O_2 and PEEP is continued as per standard treatment. If inadequate spontaneous breathing, the doctor should use the bag to support ventilation in accordance with existing guideline. INOmax at the dose of 20 ppm will be delivered for 5 minutes before any attempt to intubate (or decision not to) will be made.

- After 5 minutes RSI may be performed at the discretion of the study doctor in accordance with departmental procedure for RSI in this category of patients.
- Following RSI, the ventilation bag is squeezed the 3-4 times with O_2 / INO before starting ventilation. This is to empty the bag of any NO_2 that may have been built up.
- Regardless whether RSI is performed, or bag/mask ventilation is continued, a dose reduction of iNO should be attempted if $SpO_2 \geq 94\%$ after 5 minutes (t_1)
- Reduction is made in steps of 5 ppm every 5 minutes unless SpO_2 drops by > 5 percentage points from the value at t_1.
- In case weaning is completed during transportation, self-expandable bag ventilation may be substituted with a mechanical ventilator.
- After arrival in hospital, when patient is intubated, or a decision not to intubate has been made, iNO administered by INOBlender should be weaned by 5 ppm every 5 minutes. In the unlikely event of a rebound effect (as assessed by the study doctor) iNO may be continued temporarily as necessary. INO administrated by other devices (i.e.: not IMP) may be initiated as per local routines at the discretion of the attending doctor taking over the responsibility for the patient.

The total duration and concentration of IMP the patient has received will be recorded in the CRF.

5.3.1 INO delivery system and study setup

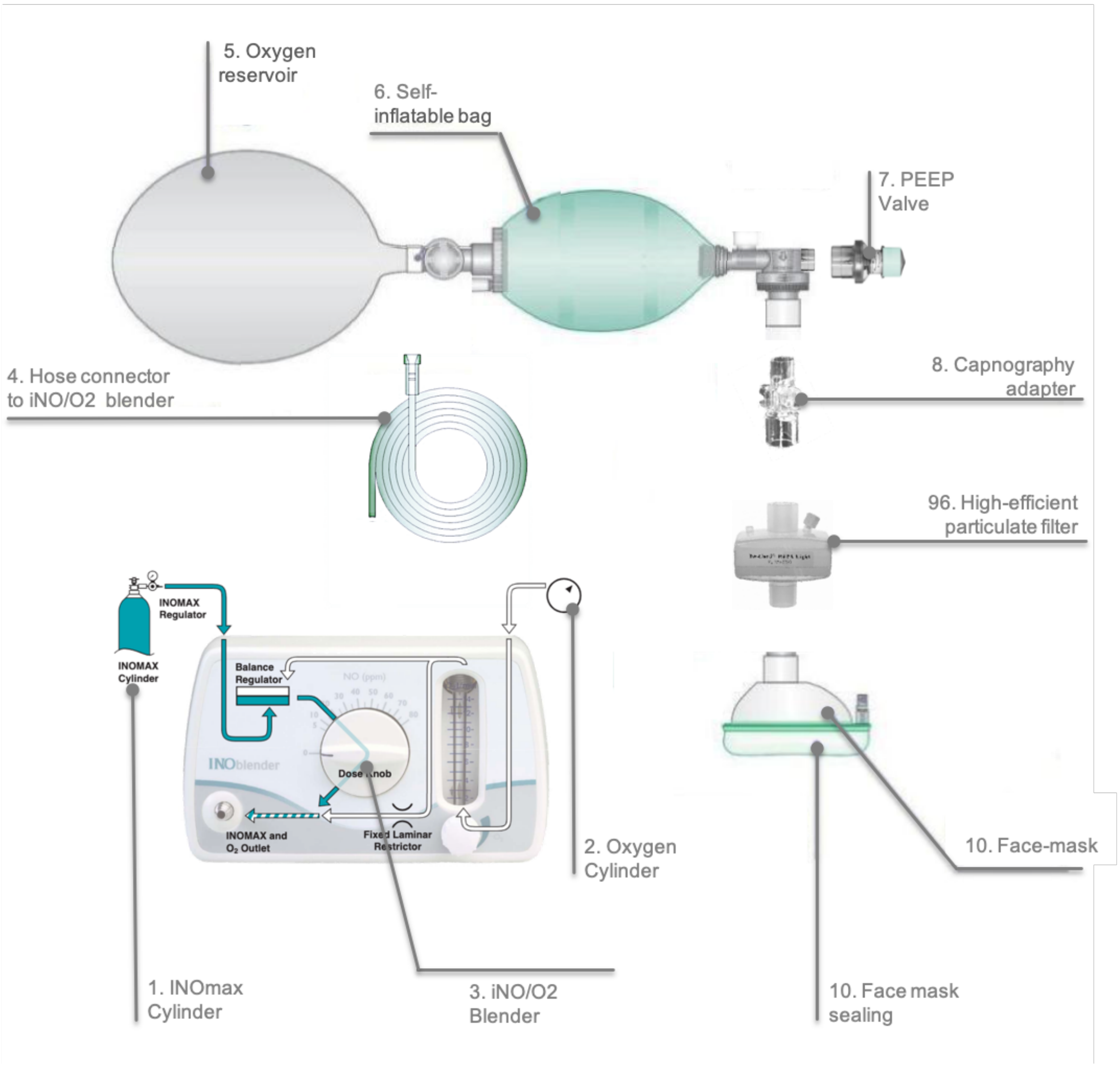

1. INOmax ® Nitric Oxide 800 ppm mol/mol, Linde Healthcare AB (EU/1/01/194/001, EU/1/01/194/002)
2. Conoxia ®, Medical Oxygen 100%, Linde Healthcare AB (N 06-4045)
3. INOBlender ® Mallinckrodt Pharmaceuticals
4. Ambu Spur II ®, Oxygen Hose, AMBU A/S
5. Ambu Spur II ®, Oxygen Reservoir, AMBU A/S
6. Ambu Spur Spur II ®, Adult Resuscitator self-expandable bag, AMBU A/S
7. Disposable PEEP 20 valve®, AMBU A/S
8. CapnoAdaptor Corpulse
9. HEPA filter
10. Face mask

The INOBlender (3) is a CE certified device for blending medical oxygen and INOmax. The blender is connected to one cylinder of CONOXIA (2) and one cylinder of INOmax (1). The INOBlender is connected to a self-inflating resuscitator bag (6), with a reservoir (5) through a dedicated tubing (4). Gas flow into the bag is diverted either to the main bag, or the reservoir. By spontaneous breathing or by squeezing the bag, gas flows from the bag and the reservoir through a flap valve in the direction of the patient. The gas flows through the in-line capnography adapter (8) and the HEPA-filter before entering the face mask (10). On Exhalation gas from the patient flows in the reverse direction and exits through the PEEP valve (7). During the study period the hospital may change suppliers of some of these components. These will be incorporated into the trial setup without applying for protocol amendments but recorded in the TMF. No changes to INOblender or INOmax is allowed without notifying competent authority. The total received dose will be calculated based on INO concentrations and duration.

5.4 Premedication and Monitoring

5.4.1 Premedication:

- Medical oxygen is mandatory in all patients included and will be recorded in the CRF
- All other medications administered by treating physician prior to IMP will be recorded in the CRF

5.4.2 Monitoring and equipment

Minimal monitoring prior to administering IMP, and recorded in the CRF is:

- Pulse oxygen saturation
- EtCO_2
- Resuscitation pads
- Blood pressure monitoring: Non-invasive or arterial line
- Vascular access: central or peripheral intravenous lines or intraosseous access

Additional monitoring if available:

- Near-infrared spectroscopy (NIRS) cerebral oxymetry
- Pulse induced continuous cardiac output (PiCCO)
- Arterial blood sampling for blood gas analysis
- Central venous pressure

5.5 Concomitant Medication

Non- IMP to all patients in this trial:

Medical oxygen (ATC: V03AN01) Medicinal gas, compressed. Current Trade Name: Conoxia 100 % . Oxygen will be delivered using standard and non-modified equipment, and connected to INOBlender as shown in 5.3.1. It will be delivered at a rate of 10 Litre/ minute. Please see attached SPC (in Norwegian)

- No medication excludes participants from being included in this trial.
- Medical oxygen will be delivered to all patients included in this trial.
- Medications used by patients prior to acute illness and inclusion in this trial will be recorded if information is available at the time of inclusion.
- All medications administered as part of emergency treatment and intubation will be recorded in the CRF. It is expected that patient will be treated with sedatives such and ketamine or propofol, opioids such as fentanyl and neuromuscular blockers such as rocuronium bromide. Medications will be recorded as ATC number, generic name, dose and route of administration. The list below is an example of the most common medications used, this is not exhaustive. A search at interaksjoner.no between these listed medications with R07A X01 Nitrogenoksid does not yield any interactions. That search also informs us that the database is not updated. The SmPC does states some caution with concomitant use of prilocaine, a local anesthetic not used in emergency medicine. This is a very unlikely happening, and if co-administered will be noted in the CRF.

Common medication in Air Ambulance setup (In Norwegian)	
Adenosin life inf/inj 5mg/ml	Lidokain mylan inj 20mg/ml
Adrenalin base NAF injkons 1mg	Magnesiumsulfat inf kons 1mmol
Adrenalin takeda inj 0,1mg/ml	Metalyse inj subst 10OO0U
Adrenalin takeda inj 1mg/ml	Midazolam braun inf/inj 1mg/ml
Aminophylline amd inj 25mg/ml	Na kl braun inf 9mg/ml Eco+
Amiodaron ham inf/inj kon 50mg	Na kl braun infkon 1mmol/ml MP
Aspirin inf/inj subst 500mg	Na kl fres t par br 9mg/ml pl
Atropin takeda inj 1mg/ml	Naloxon hamel inf/inj 0,4mg/ml
Atrovent inh væske 0,125mg/ml	Nifedipin AL kaps 10mg
Bricanyl inj 0,5mg/ml	Nitrolingual munnspr 0,4mg/dos
Calciumklorid inf kons 1mmol	Noradrenalin abc infkon 1mg/ml
Cefotaxim mip inf/inj subst 2g	Ondansetron hameln inj 2mg/ml
Curacit inj 10mg/ml	Oxytocin or inf/injkons 5 IE
Deksklorfeniramin NAF inj 5mg	Paracet stikkpille 125mg
Disprin spee disperg tab 300mg	Plavix tab 300mg
Efedrin takeda inj 50mg/ml	Propolipid inf/inj 1Omg/ml
Esmeron inj 1Omg/ml	Propolipid inf/inj 1Omg/ml
Fentanyl hameln inj 50mcg/ml	Seloken inj 1mg/ml
Flumazenil hameln inj 0,1mg/ml	Solu-Cortef inj subst 250mg
Glukose braun inj 500mg/ml	Ster vann braun t par br Eco+
Heparin leo inj 5000IE/ml	Trandate orifarm inj 5mg/ml

Isoprenalin NAF inj 0,2mg/ml	Tranexamic acid sir inj 100mg
Ketalar inj 50mg/ml	Ventoline inh væske 1mg/ml
Other medications may be added or changed at short notice without warranting changes to this protocol or notifications to competent authorities	

- Medications administered after end of Intervention will not be recorded in the CRF. Critically ill patients with long stays in the ICU such recording is beyond this trial.

5.6 Drug Accountability

The responsible site personnel will confirm receipt of IMP and will use the IMP only within the framework of this clinical study and in accordance with this protocol. Receipt, distribution and return will be documented in the TMF.

Specific systems based on the NorCRIN template for Drug accountability form and Investigational medicinal product-drug reconciliation form will be produced.

5.7 Drug Labelling

Both IMPs in this trial has a Marketing Authorization in the EU and is sourced from the EU market. They are used in the trial without modification and the packaging and labelling is carried out for local use only as per article 9.2. of the Directive 2005/28/EC (GCP Directive). We therefore apply for exemption from the specific labelling requirements for clinical trials.

5.8 Subject Numbering

Each subject is identified in the study by a unique subject number that is assigned when randomized. The subject number will match the number on the paper CRF that is found when opening the backpack holding the INOmax and the INOblender.
The number will be three digits, the first digit representing which site within the Oslo University Hospital study center.
1 denotes helicopter base Lørenskog
2 denotes physician manned ambulance 119
3 SAR helicopter based at Rygge
4 denotes Ullevål hospital Intensive care unit
5 denotes Rikshospitalet Intensive care unit
Other numbers may be added later

6 STUDY PROCEDURES

6.1.1 Before Treatment Starts

Recruiting doctor will receive a request from dispatch or the emergency department (ED). Information on SARS-COV-2 status and risk of exposure will be provided by the dispatch or the ED. This information combined with additional information obtained by the doctor prior to patient contact, determines whether the patient qualifies as confirmed, suspected or no COVID-19. Depending on all the gathered information, the doctor will decide whether to prepare the INO study kit. On first contact (t_{-1}) the patient is screened for other inclusion/exclusion criteria, according to check-list on the CRF.
If eligible, the oxygen delivery system (such as open face mask, CPAP etc.) currently used is removed, and INO treatment kit is applied with Oxygen 10l/min on sealed face mask, PEEP set to 10 cm H_2O, and INOBlender set to zero. Bag mask and PEEP valve with 100% O_2 is considered better or equal to other non-invasive measures, available to patients in the ambulance service. O_2 + 10 cmH_2O PEEP is continued for 3 minutes. If SpO_2 is <90% at this point, patient will be randomized to either INO or only O_2 (t0). If SpO_2 > 90% patients will be treated as per local guidelines outside study protocol.

6.1.2 Treatment

The doctor's assistant, who is specifically trained for the procedure, will open a sealed envelope with information on patient allocation, and either open or leave the INO cylinder closed accordingly. If randomized to only O_2 a simulated cylinder opening will be undertaken. Regardless of randomization the INOBlender is set to deliver 20 ppm. SpO_2 is recorded at time of allocation ($t_{0,}$) and after 5 minutes (t_1). Other vital signs are automatically and continuously recorded in the internal memory of the patient monitor and extracted at 5 minute intervals to EOI ($t_{0,}$ to t_n).
If the patient is included outside hospital, the doctor makes the decision to either perform RSI prior to transportation or continue current treatment during transport to hospital.
At the time the clinician decides to perform RSI, the lowest SpO_2 during the RSI-procedure is recorded (t_{RSI}) regardless of where the procedure is performed.
In any event where the bag/mask/valve is left idle, four normal tidal volumes must be ventilated into free air before re-connected to patient in order to purge the system of any NO_2 build up.
Blood samples, physiological data and clinical scoring made after EOI is done as indicated by local routines regardless of the study. Relevant clinical data is retrieved from the institution's patient journal on follow-up at day 28 post admission.

6.1.3 End of Intervention

End of intervention is defined as the time point were INO administered by the INOBlender is discontinued. This may be on different time points before transport, during transport or in hospital. For patients still requiring INO, the receiving hospital INO must be delivered by another INO device, and is no longer defined as IMP.

6.1.4 Follow up visit

Each patient will have data collected in the time from End of Intervention until day 28 after allocation. This will be done by contacting the hospital to which the patient was admitted. A set of questions assessing secondary endpoints and adverse events will be collected and recorded in the database. For patients still admitted to hospital at day 28 the study team will collect the discharge notes from main hospital admission for patients after follow-up visit and prior to end of Trial for SAE data.

6.1.5 End of Trial

End of trial is defined as the last 28 day visit of the last patient

6.1.6 Flow Chart

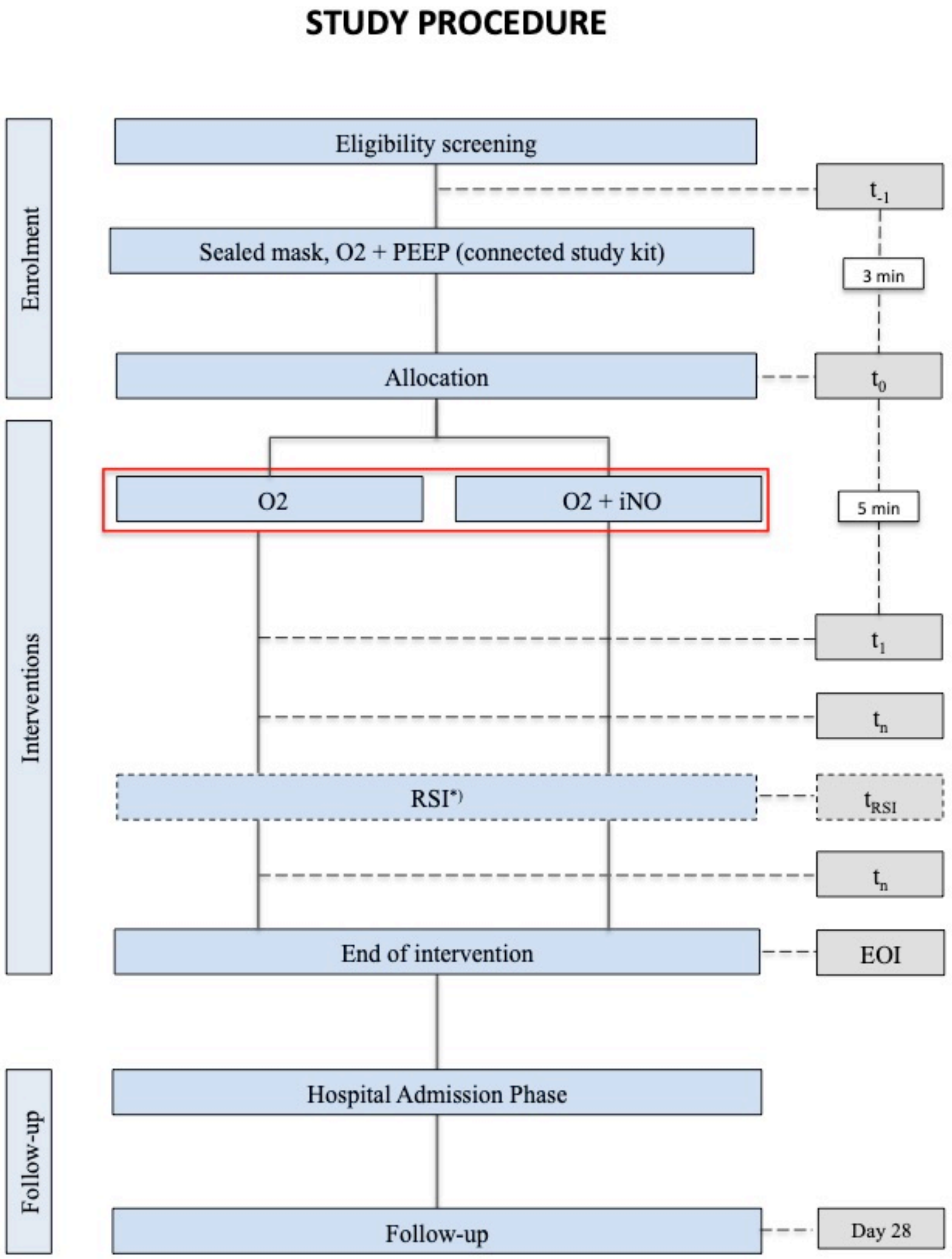

6.1.7 Schedule of Activities

Procedure	Screening	Time point (t)							E/D	Follow-up (28 days)	'Notes E/D = Early Discontinuation
		–1	0	1	n	n	n	EOI			
						RSI					
Eligibility											
-Informed consent											Variable time point
- Inclusion criteria	X										
- Exclusion criteria	X										
Airway intervention											
- PEEP + O_2		<================================>									
- RSI						X					
Allocation			X								
Study Intervention											
- INO 20 ppm			<==========================>								
Measurements (endpoint)											
- SpO_2 (1.1.1 and 1.2.1-1.2.3)	X		X	X	x	X	X	X	X		Emergency unblinding at doctor's discretion if < 50
- BP (1.2.6 and 2.1.2)			X	X	X		X	X	X		Discontinue if CA
- Respiration rate (1.2.4)			X	X	X						
- HR (1.2.6 and 2.1.1)			X	X	X		X	X	X		Discontinue if CA
- $EtCO_2$ (3.1.3)							X	X		X	
- GOS-E (1.2.7)										X	
- Mortality (1.2.8)										X	
- Se- urea concentration (2.2.1)										X	
- KDIGO (2.2.2)										X	
- Methaemoglobin (2.3.1)										X	
- Platelet count (2.4.1)										X	
- Need for INO (2.5.1)										X	
- ICD Dx (2.5.2)										X	
- O_2 ratio (3.1.1)										X	
- Se - Cardiac troponin T (4.1.1)										X	
- ProBNP (4.1.2)										X	
Sars COV 19 test										x	

Optional Measurements (endpoint)											
- art. blood gas (1.2.5 and 3.1.2)			X	X		X	X	X	X		
- Invasive art. pressure (2.1.2)			X	X		X	X	X	X		
(S)AE review			<=======================>							X	
Concomitant medication		<============================>								X	

6.2 Criteria for Patient Discontinuation

Patients will not be able to discontinue treatment prior to end of intervention in the present trial but can discontinue after end of intervention but before the 28-day follow-up.
If SpO_2 is < 50% at any point after allocation/randomization the patient case can be emergency unblinded and treatment continued as decided by study doctor. In case of cardiac arrest, INO is continued at the discretion of the study doctor. In case a serious adverse reaction triggered by IMP is suspected, an emergency blinding should be performed, and treatment with IMP discontinued or continued at the discretion of the study doctor.

6.3 Trial Discontinuation

The whole trial may be discontinued at the discretion of the PI or the sponsor in the event of any of the following:

- Occurrence of AEs unknown to date in respect of their nature, severity and duration
- Medical or ethical reasons affecting the continued performance of the trial
- Difficulties in the recruitment of patients
- At sponsors discretion

The sponsor and principal investigator will inform all investigators, the relevant Competent Authorities and Ethics Committees of the termination of the trial along with the reasons for such action. If the study is terminated early on grounds of safety, the Competent Authorities and Ethics Committees will be informed within 15 days.

6.4 Laboratory Tests

There will be no specific laboratory test performed on participants in this trial beyond standard clinical practice. There will be no biobank established.
All laboratory test in this trial will be performed in accordance with Oslo University Hospital and laboratory standard procedures.

7 ASSESSMENTS

7.1 Assessment of Efficacy

7.1.1 Primary endpoint: Oxygen saturation

Hypoxemia is one of the key features of COVID-19 related respiratory failure. Hypoxia may lead to serious brain damage, cardiac arrest and death. In susceptible patients, hypoxia may trigger acute serious diseases such as myocardial infarction and stroke. Mechanical ventilation is the mainstay in the treatment of hypoxia in COVID-19. To establish mechanical ventilation the patient needs an endotracheal tube (ETT). This is established through a rapid sequence intubation (RSI) procedure. During this procedure, the risk of severe

hypoxia is aggravated. Maintaining a higher oxygen saturation is therefore considered beneficial. Measuring SpO_2 is a direct reflection of what we aim to achieve with this treatment and is therefore chosen as the primary endpoint (1.1.1 table).
Oxygen saturation is the percentage of haemoglobin binding sites occupied by oxygen in the blood ($HbO_2/(HbO_2+Hb)$). The peripheral oxygen saturation (SpO_2) is an approximated measure of arterial oxygen saturation and is measured non-invasively by pulse oximetry. The pulse oximeter uses a light emitting diode in conjunction with a light-sensitive sensor that measures the absorption of red and infrared light in the extremity. The SpO_2 is calculated from the difference between oxygenated and deoxygenated haemoglobin. Oxygen saturation is a routinely used measurement of patient oxygenation. Sources of error such as nail polish, ambient infra-red or ultraviolet light will be considered as per normal routine.
There is no cut-off value where we can decide whether hypoxia is harmful for the patient or not. This will be dependent on other clinical features of the patient. For the primary endpoint we therefore choose to evaluate the change in SpO_2 as a continuous variable. According to general anaesthetic practice, increasing the SpO_2 to as close to 100% as possible is considered desirable prior to RSI. Thus, any increment in SpO_2 is considered beneficial. For this study we assume that an increase in SpO_2 of 5 percentage points prior to RSI is a reasonable estimate of what is clinically significant.

7.1.2 Secondary endpoints:

Primary objective
The time to effect of iNO in these patients is uncertain. For the same reasons as for the primary endpoint we have included increase in SpO_2 after 10 minutes as a secondary endpoint (1.2.1)
The overall cellular stress during hypoxemia is affected both by the degree of hypoxia and the length of the hypoxic insult. The longitudinal changes in SpO_2 during the intervention (1.2.2) is therefore a valid marker of the efficacy of the INO treatment.
Oxygen saturation during RSI (1.2.3) is a value subject to several factors that cannot be controlled for in this study. This is presumably however an important value in predicting cerebral or cardiac stress and disease. When SpO_2 falls below 50% the accuracy of measurement is low. At the same time, this value has certainly potential for causing serious brain or cardiac damage. The number of patients with this low SpO_2 therefore included as a secondary endpoint (1.2.4). Respiratory rate (1.2.5) reflects hypoxic drive and is expected to fall as SpO_2 increases. Arterial blood gas analyses (1.2.6 and 1.2.7) are not easily obtainable in the prehospital environment but is considered a more accurate reflection of oxygen saturation. Where available, this measurement will be included in the analyses. On the extreme, cardiac arrest or severe bradycardia (1.2.8) are markers of hypoxemia with potentially lethal consequences and are included in the secondary analyses. Glasgow Outcome Score Extended (GOS-E) may reflect brain damage as a result of hypoxia (1.2.9). Mortality in 24 hours (1.2.9) are may be direct result of hypoxemia in this patient group and may as such be an important marker for the efficacy of iNO. Mortality at 28 days is relevant as late complications following hypoxemia may manifest at this stage.

Secondary objectives
The endpoints of the secondary objectives reflect the known adverse effects of INO. Although this are not described after short-term administration, they will be included in this study. Both bradycardia and hypotension are possible side effects of NO. Both heart rate and blood pressure will be monitored during the intervention phase, and samples every 5 minutes are used as endpoints. (2.1.1 and 2.1.2)
All other secondary outcome parameters are collected from the patient journal.

Acute kidney injury is classified in three stages according to KDIGO. (2.2.2) A more sensitive but less specific marker of kidney function is the change in serum urea concentration. (2.2.1). Both measures are gathered on the 28-day follow-up, and the value most strongly indicative of kidney failure is used for this endpoint. Platelet count, Methaemoglobinaemia is collected from the patients EPJ.

Data from patient monitor measuring impedance (ohms) will be used to calculate respiratory rate.

7.2 Safety Considerations and Assessments

Safety will be monitored by the assessments described below as well as the collection of AEs. For details on AE collection and reporting see chapter 8. and assessment schedule refer to Flow chart in Section 6.

Rebound reactions

Following abrupt discontinuation of iNO treatment this is well recognized. Rebound reaction is manifested as increased pulmonary arterial pressure, with a subsequent drop in SpO_2. The effect is mediated through downregulation of endogenous NO production. Experimentally these changes are limited during the first couple of hours of iNO treatment(14). Moreover, at least one clinical study indicate that rebound does not occur after short term treatment of 30 minutes(15). For safety reasons, iNO in this study will be gradually weaned under careful observation. (See Ch 5.3)

Renal toxicity

Renal impairment is a well described complication to INO, although it is not described in the SPC. All previous studies in adults are in intensive care unit patients, who are exposed to INO, often over several days, and much longer than patients are exposed to IMP in the current trial. We will evaluate renal function in all included patients using the Kidney Disease: Improving Global Outcomes (KDIGO)(16)

Monitoring formation of nitrogen dioxide (NO_2)

NO_2 rapidly forms in gas mixtures containing nitric oxide and O_2, and nitric oxide may in this way cause airway inflammation and damage. When administering iNO in the ICU, monitoring of concentration of nitrogen dioxide is mandatory. As a result, NO_2 sensors are built into INO delivery systems such as INOmax DSIR® Plus Delivery Systems. These systems are made for long-term delivery of iNO in closed circuits.
The present study uses the INOblender, with no measurement of NO_2. This is accounted for in the product specification where the issue of NO_2 build up is counteracted with constant flow through the system. If the gas mix is not moving through the circuit due to absent ventilation a purge procedure is described. The manual bag should be squeezed repeatedly during use to avoid NO_2 building up in the bag.
In the present trial this will not be a big issue, as there will be constant flow through the system as iNO is started. The patient will be breathing spontaneously in a bag/mask system already.
After intubation or other for any reason the bag has been idle the bag should then be purged prior to recommencing ventilation.
There will be no measurement of NO_2 formation in this trial.

Monitoring formation of methaemoglobin

According to INOmax Highlights Of Prescribing Information (attached), the mean methaemoglobin level remained below 1% in the placebo group and in the 5 ppm and 20 ppm INOmax groups, in neonates. We expect methaemoglobin formation unlikely in our dosing range and time of administration.

Methaemoglobin will be measured upon arrival to hospital as part of the arterial blood gas analysis which will be routinely sampled in these patients. We will generally conform to the advice set out in the SPC, and measure methaemoglobin within one hour of the initiation of INOmax therapy.
The highest level of methaemoglobin measured during the treatment period of this trial will be recorded in the CRF.

Effects on platelets
According to the SPC, regular monitoring of haemostasis and measurement of bleeding time is recommended during the administration of INOmax for more than 24 hours. This falls outside of the time patients in this trial will receive INO. We do not intend to specifically analyze and assess platelet function and haemostasis in our trial.

Occupational exposure
The INOblender product specification recommendation for occupational exposure to NO and NO_2 is based on average exposure over an 8-hour time period. This exceeds the study period in the present trial. The delivery of INO in the present trial represents a low dosing (20 ppm). The delivery circuit will be closed for the majority of the time during which personnel is in contact with the system. To protect staff any pregnant staff present is an exclusion criterion in this trial.
The use in the current protocol does not deviate from current practice in Oslo University Hospital, where iNO and the INOblender is in regular use for patient care in both helicopters, ambulances and the hospital. There will be no measurement of N_2 or NO in the work environment during this trial.
For reference the Norwegian exposure limits are as follows: NO_2 8 hours exposure - 0,5 ppm, or 0,96 mg/m3; NO_2 15 minutes exposure - 1ppm, or 1,91 mg/m3; and NO xx min exposure 2 ppm, or 2,5 mg/m3(17).

7.3 Risk/ benefit balance of the trial

To balance these two concepts, we have 1) identified the risks and minimized them and 2) described potential benefits to individual subjects, groups of patients and society and attempted to enhance them.

Risks Identified and mitigating efforts

1. Research on critically ill patients: Participants included in this trial are critically ill, and require expedient and skillful medical interventions. They suffer time- critical pathology and will often be recruited outside of hospital in an environment that may be challenging to emergency medical staff.

All investigators in this s trial work with these patients and in the pre-hospital or emergency department environment on the daily basis. They are specifically trained, and all their equipment and operating procedures designed for the exact patient group recruited in this trial.

2. Delay in standard care: As described in the introduction treatment following the Airway, Breathing and Circulation principles are the core in the treatment of the patients recruited in this trial. Rapid Sequence Intubation is a key part of the management in these patients, indeed the safest possible RSI is the objective of this trial. In our design the time from arrival to the patient until allocation is 3 minutes, with additional 5 minutes of bag/ mask + PEEP after allocation to treatment arm. These 8 minutes does not constitute any considerable delay in treatment for participants included in the trail compared to patients treated outside of protocol. The 8 minutes will be filled by preparation for RSI

beyond the preoxygenation (with or without INOmax). Paramedic staff and others will use this time to do all the other preparations necessary for a safe and successful RSI:

a. The preparation of medication for sedation, analgesia and muscle relaxation- including efforts to prevent medication errors and needle-stick injury
b. The establishing and securing of intravenous of intraosseous access for medication administration
c. The preparation of equipment for RSI: laryngoscope, devise to secure endotracheal tube, suction device with extra filter for Sars COV-2 protection
d. The preparation of the patient and environment, including lifting to gurney while bag/mask ventilating, clearing out furniture or other objects blocking, evacuating patient to more suitable location, e.g. outside or inside ambulance.

3. Adverse Events to the Administration of IMP: The Marketing Authorization of the IMP does not include acute respiratory failure caused by viral infections as a therapeutic indication. Temporary administration of the IMP is however widely used in acute respiratory failure of similar etiology. No modifications will be made to the equipment. INO is generally well tolerated. Data on adverse Events in the literature generally report INOmax exposure over days or hours, while this trial propose a very short exposure. There is an extensive AE/SAE reporting procedure in placement and a DMC who monitors the trial. The most acute adverse event anticipated is a rebound reaction. All investigators are trained anaesthetist, with study specific training to recognize and handle such reaction.
4. INOmax may not work, or it may not be better than the standard treatment. In this case we believe standard care is acceptable, and that adding INOmax does not reduce the quality of standard care.

Benefit and efforts to maximize this

1. Individual participants: There is a potential benefit in receiving new treatment as part of RSI before it is available to everyone.

2. Individual participants: Knowing that the knowledge gained may benefit others in the future.

3. Transparency/ Sharing of data: The data sharing policy is important in all clinical research, particularly when a data regarding an emerging pandemic is studied, so that other may use this to increase our knowledge. This is important to maximize benefit.

The risk/ benefit balance is the representation of the principles of non- maleficence (first: do not harm) and beneficence (act to the benefit of others). It cannot be calculated from a mathematical formula, but we believe this study is designed in favour of beneficence.

8 SAFETY MONITORING AND REPORTING

The investigator is responsible for the detection and documentation of events meeting the criteria and definition of an adverse event (AE) or serious adverse event (SAE).
Please note that in the present trial all included participants are expected to meet one or more criteria for serious adverse events. The nature of the patient population studied, the prehospital environment, severity of illness included, and multitude of medical interventions makes the AE reporting challenging. Several events that meet the AE criteria set out in GCP are expected in the natural history of critical illness and in the treatment offered to patients included in this trial. On this basis we find support in the literature to prespecify outcomes that would be reported as AEs in other trials but are expected outcomes in the present. This system has previously been used in randomized trials of INO in congenital heart disease(18, 19).

8.1 Definitions

8.1.1 Adverse Event (AE)

An AE is any untoward medical occurrence in a patient administered a pharmaceutical product and which does not necessarily have a causal relationship with this treatment.
The term AE is used to include both serious and non-serious AEs.

8.1.2 Serious Adverse Event (SAE)

Any untoward medical occurrence that at any dose:

- Results in death
- Is immediately life-threatening
- Requires in-patient hospitalization or prolongation of existing hospitalization
- Results in persistent or significant disability or incapacity
- Is a congenital abnormality or birth defect
- Is an important medical event that may jeopardize the subject or may require medical intervention to prevent one of the outcomes listed above.

Medical and scientific judgment is to be exercised in deciding on the seriousness of a case. Important medical events may not be immediately life-threatening or result in death or hospitalization but may jeopardize the subject or may require intervention to prevent one of the listed outcomes in the definitions above. In such situations, or in doubtful cases, the case should be considered as serious. Hospitalization for administrative reason (for observation or social reasons) is allowed at the investigator's discretion and will not qualify as serious unless there is an associated adverse event warranting hospitalization.

8.1.3 Adverse Reaction (AR):

Events deemed to have a Certain, Probable/ Likely or Possible causal relationship of the event to IMP will be classified as an Adverse Reaction.

8.1.4 Suspected Unexpected Serious Adverse Reaction (SUSAR)

Suspected Unexpected Serious Adverse Reaction: SAR that is unexpected possibly related to INO.

8.2 Expected Adverse Events

8.2.1 Expected Adverse Events from Inhaled NO

Please consult the Summary of product characteristics for INOmax 800 ppm mol/mol medicinal gas for complete safety information.
In summary the most frequent expected AE is the rebound reaction, defined as "Abrupt discontinuation of the administration of inhaled nitric oxide may cause rebound reaction; decrease in oxygenation and increase in central pressure and subsequent decrease in systemic blood pressure. Rebound reaction is the most commonly adverse reaction in association with the clinical use of INOmax. The rebound may be seen early as well as late during therapy."
The duration of therapy of INOmax in this SPC is up to 7 days in the peri-operative setting, and common treatment times are 24 -48 hours. In the present trial the treatment time is expected to be substantially shorter, we expect this to reduce the risk of all adverse events.

Summary Of Product Characteristics 4.8 Undesirable effects						
System organ class	Very common	Common	Uncommon	Rare	Very rare	Not known
Blood and lymphatic system disorders	Thrombo-cytopenia	-	Methemoglobinemia	-	-	-
Cardiac disorders	-	-	-	-	-	Bradycardia (following abrupt discontinuation of therapy)
Vascular disorders	-	Hypotension	-	-	-	-
Respiratory, thoracic and mediastinal disorders	-	Atelectasis	-	-	-	Hypoxia<br>Dyspnoea<br>Chest Discomfort<br>Dry throat
Nervous system disorders	-	-	-	-	-	Headache<br>Dizziness

8.2.2 Other Expected Serious Adverse Events in current trial

Patients in the current trial are critically ill and are included early in what is expected to be a prolonged hospital stay, often in intensive care unit and with several interventions over many days or even weeks. Both the standard care prior to arrival in hospital and during their stay have significant risks for adverse events. Below is a table of expected serious adverse events. These will be recorded in the CRF, but not reported to Sponsor, see flow chart section 8.5.

Expected Serious Adverse Events	
System Organ Class (MedDRA)	Expected event
Blood and lymphatic system disorders	• Methemoglobinemia
Cardiac disorders	• Cardiac arrest<br>• Myocardial infarction<br>• Ventricular fibrillation<br>• Ventricular tachycardia<br>• Bradycardia
Immune system disorders	• Acute anaphylaxis
Infections and infestations	• Acute infection
Injury, poisoning and procedural complications	• Iatrogenic pneumothorax<br>• Incision site bleeding<br>• Anaesthesia intubation complication
Nervous system disorders	• Brain hypoxia
Renal and urinary disorders	• Hypoxic kidney injury<br>• Renal failure
Respiratory, thoracic and mediastinal disorders	• Aspiration<br>• Pneumonia<br>• Pneumothorax<br>• ARDS<br>• ALI
Skin and subcutaneous tissue disorders	• Pressure wounds
Surgical and medical procedures	Events classified under MedDRA high level term:<br>• Anaesthesia and allied procedures<br>• Venous therapeutic procedures<br>• Arterial therapeutic procedures<br>• Therapeutic procedures NEC
Vascular disorders	• Acute hypotension

Please note: Both suspected rebound reactions and death prior to end of intervention will always be reported to Sponsor as a serious adverse event.

8.3 Time Period for Reporting AE and SAE

- For each patient the standard time period for collecting and recording AE will be from allocation and until End of Intervention
- For each patient the standard time period for collecting and recording SAE will be from allocation and until the 28 day follow up visit. At the 28 days visit an assessment will be made where we specifically ask for all SAEs mentioned in chapter 8.2. We will also assess discharge notes and treating physician for other events that fulfil the SAE definition and that can be assessed by the study team.
- For patients still admitted to hospitals at day 28 an effort will be made to secure access to main discharge note for assessment for any SAE that has occurred between day 28 and discharge.
- Any information that comes to investigators attention for any included patient prior to End of Trial will be assessed according to this protocol

8.4 Recording of Adverse Events

If the patient has experienced adverse event(s), the investigator will record the following information in the CRF:

- The nature of the event(s) will be described by the investigator in precise standard medical terminology
- The duration of the event will be described in terms of event onset date and event ended date, if known

Severity of Adverse Events
All AEs and SAEs will be assessed for severity, according to the Division of AIDS (DAIDS) Table for Grading the Severity of Adult and Paediatric Adverse Events, version 2.1 (July 2017).
The following guidelines will be used to describe severity.

Mild (Grade 1): Events that are usually transient and may require only minimal or no treatment or therapeutic intervention and generally do not interfere with the subject's usual activities of daily living.
Moderate (Grade 2): Events that are usually alleviated with additional specific therapeutic intervention. The event interferes with usual activities of daily living, causing discomfort but poses no significant or permanent risk of harm to the research subject.
Severe (Grade 3): Events interrupt usual activities of daily living, or significantly affects clinical status, or may require intensive therapeutic intervention. Severe events are usually incapacitating.
Severe (Grade 4): Events that are potentially life threatening.

- The Causal relationship of the event to the study medication/IMP will be assessed as one of the following according to the WHO-UMC system for standardized case causality assessment(20):

Causality term	Assessment criteria*
Certain	• Event with plausible time relationship to drug intake<br>• Cannot be explained by disease or other drugs<br>• Response to withdrawal plausible (pharmacologically, pathologically)<br>• Event definitive pharmacologically or phenomenologically (i.e. an objective and specific medical disorder or a recognized pharmacological phenomenon)<br>• Rechallenge satisfactory, if necessary
Probable/ Likely	• Event with reasonable time relationship to drug intake<br>• Unlikely to be attributed to disease or other drugs<br>• Response to withdrawal clinically reasonable<br>• Rechallenge not required
Possible	• Event with reason able time relationship to drug intake<br>• Could also be explained by disease or other drugs<br>• Information on drug withdrawal may be lacking or unclear
Unlikely	• Event with a time to drug intake that makes a relationship improbable (but not impossible)<br>• Disease or other drugs provide plausible explanations
Conditional/ Unclassified	• Event or laboratory test abnormality<br>• More data for proper assessment needed, or<br>• Additional data under examination
Unassessable/ Unclassifiable	• Report suggesting an adverse reaction<br>• Cannot be judged because information is insufficient or contradictory<br>• Data cannot be supplemented or verified

- Action taken
- The outcome of the adverse event – whether the event is resolved or still ongoing
- AEs will be coded according to the MedRA system by medical monitor

8.5 Reporting Procedure

Then flow chart describes the SAE reporting procedure:

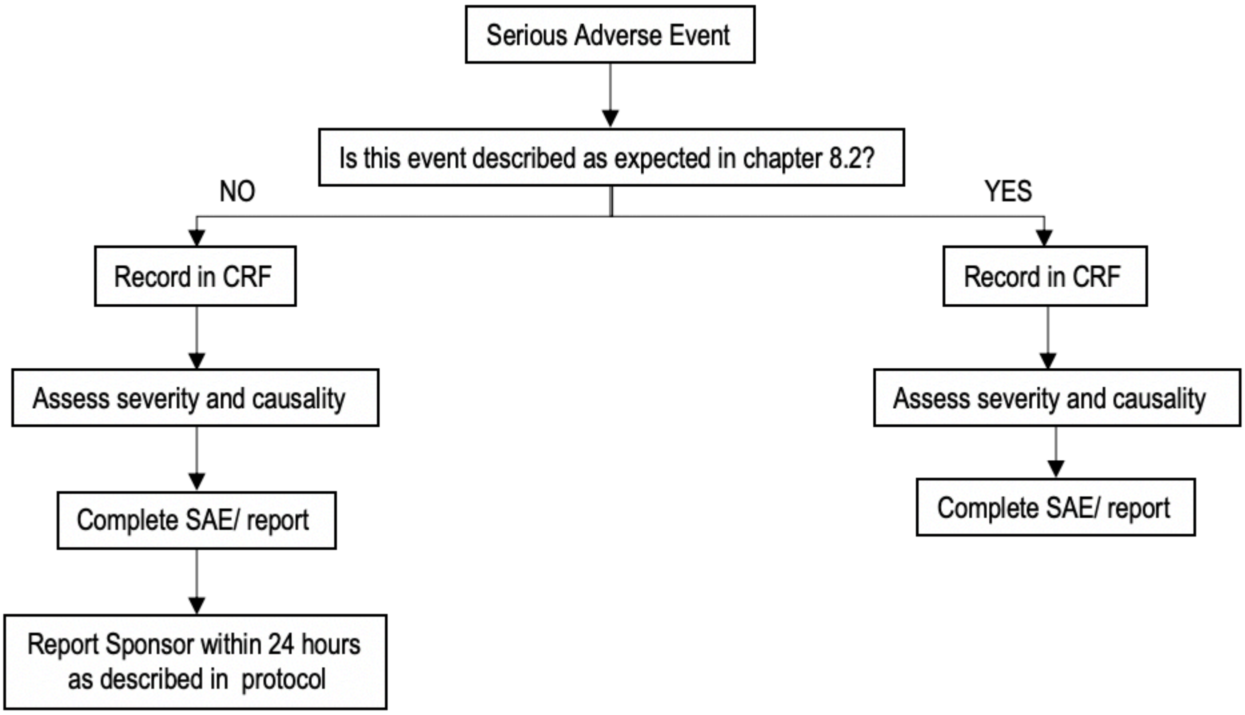

Unexpected SAEs must be reported by the investigator to the sponsor, Christian Buskop, using the Oslo University Hospital reporting system Achilles, within 24 hours after the site has gained knowledge of the unexpected SAE. Sponsor should also be notified via telephone od SMS at 99438374 when a report is sent.

Every SAE must be documented by the investigator on the SAE pages as part of the CRF. The Serious Adverse Event Report Form must be completed, signed and sent to Hans Julius Heimdal. The initial report shall promptly be followed by detailed, written reports if necessary. The initial and follow-up reports shall identify the trial subjects by unique code numbers assigned to the latter.

The sponsor keeps detailed records of all SAEs reported by the investigators and performs an evaluation with respect to seriousness, causality and expectedness.

8.5.1 SUSARs

SUSARs will be reported to the Competent Authority and Ethics Committee according to national regulation. The following timelines should be followed:
The sponsor will ensure that all relevant information about suspected serious unexpected adverse reactions that are fatal or life-threatening is recorded and reported as soon as possible to the Competent Authority and Ethics Committee in any case no later than seven (7) days after knowledge by the sponsor of such a case, and that relevant follow-up information is subsequently communicated within an additional eight (8) days.
All other suspected serious unexpected adverse reactions will be reported to the Competent Authority concerned and to the Ethics Committee concerned as soon as possible but within a maximum of fifteen (15) days of first knowledge by the sponsor.
SUSARs will be reported using the CIOMS form since Oslo University Hospital is not connected to EudraVigilance.

8.5.2 Annual Safety Report

Once a year throughout the clinical trial, the sponsor will provide the Competent Authority with an annual safety report. The format will comply with national requirements.

8.5.3 Clinical Study Report

The adverse events and serious adverse events occurring during the study will be discussed in the safety evaluation part of the Clinical Study Report. The report will be sent to the Norwegian Medicines Agency within one year of the completion or discontinuation of the trial.

8.6 Procedures in Case of Emergency and unblinding

The principal investigator is responsible for assuring that there are procedures and expertise available to cope with emergencies during the study prior to including patients in this trial.

For medical emergencies during treatment in this trial the standard operating procedures for the participating department will be followed. For pre-hospital sites this includes telephone or radio contact with colleague via AMK, with the potential for additional recourses to be dispatched to the scene.

For participating departments in-hospital this includes calling on help from senior aides or other recourses via normal hospital procedures.

If the study doctor considers iNO treatment mandatory or contra indicated for patient benefit, he or she may perform emergency unblinding. This is done by a closed loop communication procedure in which the study doctor clearly states an intention of unblinding as well as the reason for unblinding. The assistant repeats back to the study doctor for confirmation, before revealing the allocation. The reason for emergency unblinding is recorded in the CRF as soon as practicably possible. The emergency blinding procedure will be described in the written instructions for the study doctor and the assistant.

8.7 Data Monitoring Committee (DMC)

A DMC will be recruited. Their work will be described in a charter based on the NORCRIN Standard Operating Procedure. This describes the roles and responsibilities of the DMC, including the timing of meetings, methods of providing information to and from the DMC, frequency and format of meetings, statistical issues and relationships with other Sponsor. The charter and the DMC should be in place before the first patient is included.

The DMC will meet after the interim analysis, and in the event of SUSAR reporting.

9 DATA MANAGEMENT AND MONITORING

Data management will be performed by the data management unit at the Clinical Trials Unit, Oslo University Hospital. The Data management procedures will be performed in accordance with the department's SOPs and ICH guidelines. The data management process will be described in the study specific Data Handling Plan and the study specific Data Handling Report after database closure.

Data entered into the eCRF will be validated as defined in the Data Validation Plan. Validation includes, but is not limited to, validity checks (e.g. range checks), consistency checks and customized checks (logical checks between variables to ensure that study data are accurately reported) for eCRF data and external data (e.g. laboratory data). A majority of edit checks will be triggered during data entry and will therefore facilitate efficient 'point of entry' data cleaning.

Data management personnel will perform both manual eCRF review and review of additional electronic edit checks to ensure that the data are complete, consistent and reasonable. The electronic edit checks will run continually throughout the course of the study and the issues will be reviewed manually online to determine what action needs to be taken.

Manual queries may be added to the system by clinical data management or study monitor. Clinical data managers and study monitors are able to remotely and proactively monitor the patient eCRFs to improve data quality.

All updates to queried data will be made by authorized study center personnel only and all modifications to the database will be recorded in an audit trail. Once all the queries have been resolved, eCRFs will be signed by electronic signature. Any changes to signed eCRFs will be approved and resigned by the Investigator.

Adverse events will be coded from the verbatim description (Investigator term) using the Medical Dictionary for Regulatory Activities (MedDRA).

Once the full set of eCRFs have been completed and locked, the Sponsor will authorize database lock and all electronic data will be sent to the designated statistician for analysis. Subsequent changes to the database will then be made only by written agreement.

The data will be stored in a dedicated and secured area at Oslo University Hospital. Data will be stored in a de-identified manner, where each study participant is recognizable by his/her unique trial subject number. The data will be stored until 15 years following the last patient's final study visit.

9.1 Case Report Forms (CRFs)

Two paper CRFs will be produced.
1: For treatment visit, this will capture data on primary end point, length of intervention and details regarding IMP administration, protocol adherence and main safety data not captured in other source data. This will be filled in by treating doctor/ investigator administering IMP

2: day 28 visit
This will be filled out by investigator if patient admitted to OUH, or by treating doctor in cooperation with investigator if patient admitted to hospital outside OUH. This will capture secondary and explorative endpoint as described earlier, including safety data.

9.2 Source Data

Source data in this trial includes study specific paper case report form. In addition, all patient records may be sources of specific information. For patients in this trial this includes:

- Study specific paper CRF inclusion visit and day 28 visit
- Prehospital paper records, computer notes in LABAS, records in AMIS and sound or in medical dispatch system.
- Records from medical monitors such as LP15 and CorPulse
- In-hospital results in DIPS or other EPJ
- Interview with study workers or treating physician in hospital

	**Objectives**		**Endpoints**	**Source data with hierarchy if inconsistent data between sources**
	Primary		Primary	
1	Evaluate the clinical efficacy of iNO to increase oxygen saturation prior to, during and after emergency RSI in patients with suspected or confirmed COVID-19 respiratory failure.	1.1.1	ΔSpO_2	CRF > Patient monitor > Patient paper chart > Patient EPJ
			Secondary	
		1.2.1	Δ SpO_2 (t_0-t_2)	CRF > Patient monitor > Patient paper chart > Patient EPJ
		1.2.2	SpO_2 during RSI (t_{RSI})	CRF > Patient monitor > Patient paper chart > Patient EPJ
		1.2.3	SpO_2 from t_1 - t_n	Patient monitor > Patient chart > Patient EPJ
		1.2.4	Number of patients with SpO_2<50 from t_1 to EOI	CRF > Patient monitor > Patient paper chart Follow up visit CRF

			1.2.5	Respiratory rate from t_0 to t_{RSI} or EOI	Patient monitor > Patient paper chart > Patient EPJ
			1.2.6	PaO_2 at t_0 to EOI	Follow up visit CRF > Patient EPJ
			1.2.7	Cardiac arrest during intervention	CRF
			1.2.8	GOS-E score on day 28	Follow up visit CRF > Patient EPJ
			1.2.9	Mortality	Follow up visit CRF > Patient EPJ
	Secondary			Secondary	
2	Evaluate the safety of the intervention as compared to the control as assessed by:	2.1 Circulatory function	2.1.1	Heart rate at t_0-t_n	Patient monitor > Patient chart > Patient EPJ
			2.1.2	Blood pressure at t_0 to t_n	Patient monitor > Patient chart > Patient EPJ
		2.2 Kidney function	2.2.1	Increase in Serum urea concentration	Follow up visit CRF > Patient EPJ
			2.2.2	KDIGO AKI Stage up to day 28	Follow up visit CRF > Patient EPJ
		2.3 Hemoglobin function	2.3.1	Arterial MetHgb concentration	Follow up visit CRF > Patient EPJ
		2.4 Platelet count	2.4.1	Platelet count	Follow up visit CRF > Patient EPJ
			2.5.1	Need for iNO after EOI	Follow up visit CRF > Patient EPJ
			2.5.2	ICD-10 diagnosis on discharge	Follow up visit CRF > Patient EPJ
	Exploratory			Exploratory	
3.	Evaluate the clinical efficacy of iNO compared to standard treatment on respiratory severity and length of specialized care		3.1.1	O_2-ratio for patients on mechanical ventilation first 24 hours	Follow up visit CRF > Patient EPJ
			3.1.2	$PaCO_2$ at t_0 to EOI	Follow up visit CRF > Patient EPJ
			3.1.3	$EtCO_2$ at t_0 to EOI	CRF > Patient monitor > Patient paper chart > Patient EPJ
			3.1.4	Ventilator free days up to day 28	Follow up visit CRF > Patient EPJ
			3.1.5	Length of stay in ICU	Follow up visit CRF > Patient EPJ

4	Evaluate the effect of INO on cardiac stress	4.1.1	Serum troponin T	Follow up visit CRF > Patient EPJ
		4.1.2	ProBNP	Follow up visit CRF > Patient EPJ
	Patient Demographic variables			Patient EPJ
	Temporal and technical data regarding emergency dispatch			AMIS > Patient paper chart

9.3 Study Monitoring

The investigator will be visited on a regular basis by the Clinical Study Monitor, who will check the following:

- Informed consent process
- Reporting of adverse events and all other safety data
- Adherence to protocol
- Maintenance of required regulatory documents
- Study Supply accountability
- Data completion on the CRFs including source data verification (SDV).

The monitor will review the relevant CRFs for accuracy and completeness and will ask the site staff to adjust any discrepancies as required.

Sponsor's representatives (e.g. monitors, auditors) and/or competent authorities will be allowed access to source data for source data verification in which case a review of those parts of the hospital records relevant to the study may be required.

9.4 Confidentiality

The investigator shall arrange for the secure retention of the patient identification and the code list following the guidelines set out by Oslo University Hospital and its Data Protection Officer. Patient files shall be kept for the maximum period of time permitted by each hospital. The study documentation (CRFs, Site File etc.) shall be retained and stored during the study and for 25 years after study closure. All information concerning the study will be stored in a safe place inaccessible to unauthorized personnel.

10 STATISTICAL METHODS AND DATA ANALYSIS

10.1 Determination of Sample Size

One previous study in a different setting has shown that 71% of patients respond to the treatment with an absolute increase in SpO_2 of >5 percentage points(21). We assume a standard deviation of 10 percentage points, and a within subject correlation of 0.8 based on clinical experience. This implies a standard deviation of the change in SpO_2 (before minus after intervention) of 6.32%. A difference in improvement in SpO_2 of 5 percentage points between the intervention groups is considered clinically significant. To identify a difference of improvement of SpO_2 of 5 percentage points, with a power of 80%, we need to include 54 patients (27 in each arm) in this trial, using a significance level of 5%. Because of the uncertainty related in the magnitude of the variance in the SpO_2 change, a simple blind re-estimation of the sample size based on the variance from a subsample of the 20 first patients will be done. The sample size will be adjusted if necessary, but the total sample will not be reduced (to less than 54). By using a blinded one-sample variance estimator for sample size recalculation, the effect of sample size adjusting on the type I error rate is negligible and no additional measures are needed to control the significance level(22).

10.2 Randomization and Blinding

Eligible patients will be allocated in a 1:1 ratio between the two study arms, using a computer-based randomization procedure. The randomization will be blocked using varying block sizes. The randomization will be stratified between the study sites. Initially two study sites (Lørenskog and Ullevål) include patients for the study. If further sites are added during the trial, these will be treated as separate strata in the randomization.

The randomization list will be sent to the OUH Hospital Pharmacy division for clinical trial who will pack envelopes with labels indication allocation groups. The envelopes will not be able to see or light through, or open without breaking the seal. Sealed envelopes will be collected by the study team for placement on the bags contains IMP. An accountability form for all envelopes produces will be in place. Unopened envelopes will be returned to the Pharmacy.

The sealed envelope produced by the pharmacy will be placed in the iNO study kit. When a patient is found eligible, the envelope is opened by the study doctor's assistant. He or she will either open or mimic opening the INOmax cylinder according to allocation. The INOmax cylinder is contained in a bag, and the cylinder valve with the pressure gauge will not be visible to the study doctor.

The procedure of blinding and emergency unblinding will be described in a written instruction for both the study doctors and the assistants. Emphasis will be put on instructing the assistant not to take part in any study-relevant clinical decision-making or discussion about the clinical condition of the patient, including the option of emergency unblinding after randomization.

After arrival in hospital, after the patient is intubated or a decision is made not to perform intubation, the assistant will hand over the envelope containing the randomization as a part of the written treatment documentation. This is in the interest of further hospital patient care, and to evaluate additional need for INO other than IMP. The allocation will thus be revealed to the personnel taking over responsibility for the patient,

but not to the study doctor. The study doctors are specifically instructed not to try to obtain information on allocation.

10.3 Population for Analysis

The following populations will be considered for the analyses:

- Intention to treat (ITT) population: All randomized participants, regardless of protocol adherence.
- Full Analysis Set (FAS): All randomized patients where a sealed facemask has been applied with the INOBlender set to 20 ppm at any point of time.
- Per Protocol (PP) set: All randomized patients who sufficiently comply with the protocol. Criteria for inclusion in the PP population will be specified in the statistical analysis plan, and the final criteria will be defined prior to database lock.
- Safety population: All randomized patients where a sealed facemask has been applied with the INOBlender set to 20 ppm at any point of time, i.e. identical to the FAS.

The primary analysis will be done on the Full Analysis Set.

10.4 Planned analyses

There will be one safety interim analysis in this trial, and one main efficacy analysis at the end of the trial. In addition, there will be a blinded evaluation of the variance in the primary endpoint after 20 patients.

- There will be a blinded evaluation of the variance in the primary endpoint after 20 patients, and a re-estimation of the sample size. The total sample size of the trial will be either the sample size calculated prior to the start of the trial, or the re-estimated sample size, whichever is largest. Thus, the total sample size will not be reduced due to the re-estimation.
- The safety interim analysis will be performed when approximately half of the patients have completed the intervention. This will thus occur when a minimum of 27 patients have completed the intervention. In case of an increased sample size due to the sample size re-estimation, the safety interim analysis will take place when approximately half of the patients in the (total) re-estimated sample size have completed the intervention. A separate document (the Data Monitoring Committee (DMC) charter) will detail the procedures for the interim analysis. A report will be written following the interim analysis, describing any deviations from the planned analysis, and a recommendation to either continue or stop the study. Treatment efficacy will not be investigated in the interim analysis.
- The final analysis will be performed when the planned number of patients (54, or the number of patients determined by the sample size re-estimation) have completed the intervention, and all data have been entered, verified and validated according to the data management plan.

10.5 Statistical Analysis

Demographics and other baseline characteristics, such as age, gender etc., will be described for all randomized patients (the ITT population). Continuous variables will be described in terms of number of observations (n), mean value and standard deviation. All variables of a categorical nature will be displayed with frequencies and percentages. The tabulation of the descriptive statistics will be split by treatment group.

The primary efficacy endpoint, the difference in the change in SpO_2 from before to after the intervention, will be analyzed using a linear regression model where the intervention variable will be adjusted by the baseline SpO_2 and the stratification variable used in the randomization (study site). Currently there are two sites in this trial. If additional sites are added during the trial, these will represent additional strata. If the residuals from the regression model is not normally distributed, suitable transformations will be applied to the data or non-parametric alternatives will be used.

For the primary endpoint, a subgroup analysis will be performed according to later confirmed Covid-19 status of the patients. Presence of COVID-19 disease is classified as per WHO definition of confirmed COVID-19 (see ch 1.1.1), and with the SARS-CoV-2 test routine used at the given point of time in the respective institution. Data will be extracted from the EPJ on the 28-day visit.

Analyses of secondary endpoints will be performed in a similar manner as for the primary endpoint and/or using repeated measures mixed models for continuous variables, and logistic regression models for binary endpoints. Subgroup analyses will be performed according to later confirmed Covid-19 diagnosis for the secondary endpoints related to SpO_2 (endpoints 1.2.1-1.2.4).

The primary and secondary analyses will be performed in the FAS, and robustness analyses will be performed in the PP population. A separate statistical analysis plan (SAP) will provide further details on the planned statistical analyses. The SAP will be finalized, signed and dated prior to database lock. The treatment allocation will be revealed after the database lock and used in the statistical analysis.
Deviation from the original statistical plan will be described and justified in the Clinical Study Report. Amendments to plan can be done until day of database (DB) lock.

11 STUDY MANAGEMENT

11.1 Trial Master File (TMF)

A paper- based Trial Master File will be kept at the Air Ambulance Base at Lørenskog, with Invest Site Files at participating departments. These will be based on NorCrin SOP. This will made available to monitor and authorities upon request.

11.2 Investigator Delegation Procedure

The principal investigator is responsible for making and updating a “delegation of tasks” listing all the involved co-workers and their role in the project. He will ensure that appropriate training relevant to the study is given to all of these staff, and that any new information of relevance to the performance of this study is forwarded to the staff involved. A delegation log will be produced and stored in the TMF.

11.3 Protocol Adherence

Investigators ascertain they will apply due diligence to avoid protocol deviations.
All significant protocol deviations will be recorded and reported in the Clinical Study Report (CSR)

11.4 Study Amendments

If it is necessary for the study protocol to be amended, the amendment and/or a new version of the study protocol (Amended Protocol) must be notified to and approved by the Competent Authority and the Ethics Committee according to EU and national regulations.

11.5 Audit and Inspections

Authorized representatives of a Competent Authority and Ethics Committee may visit the center to perform inspections, including source data verification. Likewise, the representatives from sponsor may visit the center to perform an audit. The purpose of an audit or inspection is to systematically and independently examine all study-related activities and documents to determine whether these activities were conducted, and data were recorded, analyzed, and accurately reported according to the protocol, Good Clinical Practice (ICH GCP), and any applicable regulatory requirements. The principal investigator will ensure that the inspectors and auditors will be provided with access to source data/documents.

12 ETHICAL AND REGULATORY REQUIREMENTS

The study will be conducted in accordance with ethical principles that have their origin in the Declaration of Helsinki and are consistent with ICH/Good Clinical Practice and applicable regulatory requirements. Registration of patient data will be carried out in accordance with national personal data laws.

12.1 Ethics Committee Approval

The study protocol, including the patient information and informed consent form to be used, must be approved by the regional Ethics Committee before enrolment of any patients into the study.
The investigator is responsible for informing the Ethics Committee of any serious and unexpected adverse events and/or major amendments to the protocol as per national requirements.

12.2 Other Regulatory Approvals

The protocol will be submitted and approved by the Norwegian Medicines Agency prior to study start.

12.3 Informed Consent Procedure

The inclusion criteria in this study is hypoxia to an extent representing a severe medical condition. Therefore, at the time of inclusion, the patient is neither competent to consent, nor able to receive any information about the study. The clinical setting in which the study is performed is by nature a situation where consent prior to inclusion and treatment is not possible to obtain. It would also be ethical questionable to trust that any

information would be received and processed by the patient. Therefore, the study participants or next of kin will be informed and asked to participate post treatment. The informed consent procedure is as follows:

- Inclusion, treatment and data recording will occur before any informed consent is obtained
- After hospitalization, and within the 28 days to follow-up, either the patient (if deemed competent) or the next of kin will be informed with written information (see patient consent form) and asked to participate
- The consent will be for storing, analyzing and publish data collated in the CRF (treatment is already administered)
- If the consent is given by next of kind of a deceased patient, or a patient deemed to not recover to consent competency, the consent from next of kin is considered to be final
- If the consent is given by next of kin of a patient suspected to recover from incompetency, the consent from the patient will be obtained after recovery
- Information about the study and consent is given both oral (study personnel) and written (Patient consent form)
- Consent is written (patient consent form). Oral consent can be obtained but require signature from two health care personnel (of which one study personnel/ investigator) stating that the patient has consented.

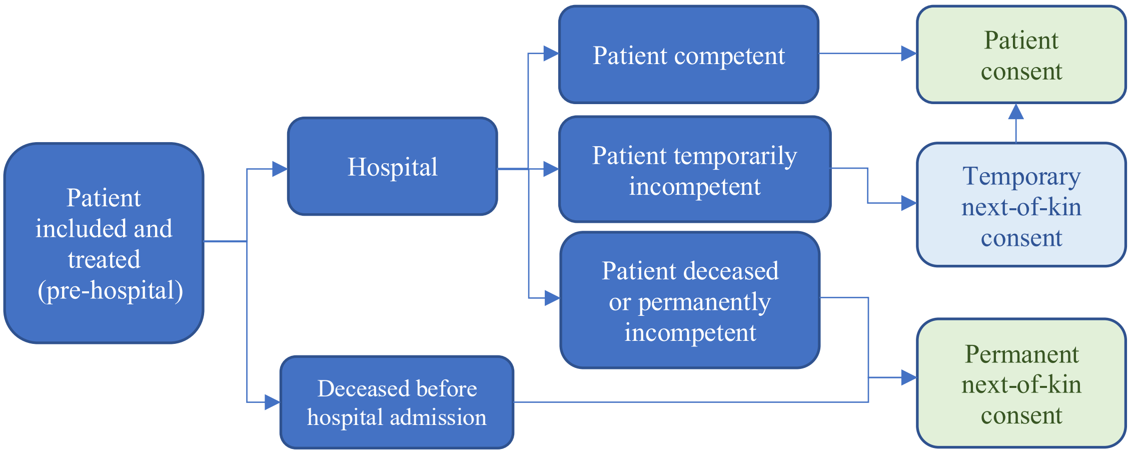

Patient consent procedure

12.4 Subject Identification

The investigator is responsible for keeping a list of all patients (who have received study treatment or undergone any study specific procedure) including patient's date of birth, personal number and full names in a code list approved storage at site for such information.
The patients will be identified in the CRFs by their three-digit randomization number.

13 TRIAL SPONSORSHIP, OWNERSHIP AND FINANCING

Oslo University Hospital retains full ownership to all data and results generated in this trial, and it is the sole financial sponsor. Further funding may be sought during the trial period. Several of the investigators are partly

employed by the Norwegian Air Ambulance Foundation. This foundation has had no place in the design or ownership in this trial.

14 TRIAL INSURANCE

The Principal investigator will obtain insurance coverage for this study through membership of the Drug Liability Association, and ensure proof of membership is sent Competent Authority and stored in the TMF prior to inclusion of the first patient.

15 PUBLICATION POLICY

The study will be sought published in a peer-reviewed scientific journal. Decision on authorships will follow the "Uniform Requirements for Manuscripts Submitted to Biomedical Journals" (the Vancouver Convention) by The International Committee of Medical Journal Editors (ICMJE).
The results of this study will also be submitted to the Competent Authority and the Ethics Committee according to EU and national regulations. Sponsor commits to publish the data, regardless of outcome.

16 DATA SHARING

OUH has complete ownership of all data and publishing rights of all results.

Metadata:
The full protocol, Statistical Analysis Plan, information letter for consent and other trial documents will be published open access. The Clinical Study report and Statistical analysis report will also be made openly available, but may be altered to hide information that may lead to identification of individual study participants. These documents will be shared at Norwegian Centre for Research Data (NSD).
NSD is a corporation owned by the Ministry of Education and Research and is is a national archive and center for research data.

Individual participant data:
All of the individual participant data collected during the trial, after de- identification will be made available to anyone who wishes to access the data. Data will be made indefinitely available through Norwegian Centre for Research Data (NSD). De- identified data can only be distributed in accordance with the data processor agreement entered into between the Sponsor and NSD.

Data sharing with editors or peer- reviewers of scientific journals, conferences or the like will not require specific consent or data access agreement with Oslo University Hospital, in the understanding that the data will not be shared onward or used beyond reviewing this trial.

After data sharing Oslo University Hospital, Pre-Hospital Division must be acknowledged in any publication resulting from the shared data. For closer collaboration authorship based on the the Vancouver Convention must be considered.

17 LIST OF APPENDICES

1. INOmax 800 ppm mol/mol medicinal gas, compressed, Summary of product characteristics
2. Conoxia 100 % , Summary of product characteristics (In Norwegian)
3. WHO; Global surveillance for COVID-19caused by human infection with COVID-19 virus Interim guidance 20 March 2020
4. WHO; Clinical management of severe acute respiratory infection (SARI) when COVID-19 disease is suspected. Interim guidance 13 March 2020
5. INOmax® - nitric oxide gasINO Therapeutics, Highlights Of Prescribing Information
6. INOblender Operation and Maintenance Manual

18 REFERENCES

1. Sun P, Lu X, Xu C, Sun W, Pan B. Understanding of COVID-19 based on current evidence. J Med Virol. 2020.
2. World Health Organization. WHO Director-General's opening remarks at the media briefing on COVID-19 - 11 March 2020 2020 [cited 2020 02.04]. Available from: https://www.who.int/dg/speeches/detail/who-director-general-s-opening-remarks-at-the-media-briefing-on-covid-19---11-march-2020.
3. Wu C, Chen X, Cai Y, Xia J, Zhou X, Xu S, et al. Risk Factors Associated With Acute Respiratory Distress Syndrome and Death in Patients With Coronavirus Disease 2019 Pneumonia in Wuhan, China. JAMA Intern Med. 2020.
4. Levy SD, Alladina JW, Hibbert KA, Harris RS, Bajwa EK, Hess DR. High-flow oxygen therapy and other inhaled therapies in intensive care units. The Lancet. 2016;387(10030):1867-78.
5. Creagh-Brown BC, Griffiths MJ, Evans TW. Bench-to-bedside review: Inhaled nitric oxide therapy in adults. Crit Care. 2009;13(3):221.
6. Griffiths MJ, Evans TW. Inhaled nitric oxide therapy in adults. N Engl J Med. 2005;353(25):2683-95.
7. Gebistorf F, Karam O, Wetterslev J, Afshari A. Inhaled nitric oxide for acute respiratory distress syndrome (ARDS) in children and adults. Cochrane Database Syst Rev. 2016(6):CD002787.
8. Gropper MA, Miller RD. Miller's anesthesia. Ninth edition.
International edition. ed. Philadelphia, PA: Elsevier; 2020. 2 volumes (xxvii, 2824, i114 pages) p.
9. Bredmose PP, Buskop C, Lomo AB. Inhaled nitric oxide might be a contributing tool for successful resuscitation of cardiac arrest related to pulmonary hypertension. Scand J Trauma Resusc Emerg Med. 2019;27(1):22.
10. Ruan SY, Huang TM, Wu HY, Wu HD, Yu CJ, Lai MS. Inhaled nitric oxide therapy and risk of renal dysfunction: a systematic review and meta-analysis of randomized trials. Crit Care. 2015;19:137.
11. Claesson J, Freundlich M, Gunnarsson I, Laake JH, Moller MH, Vandvik PO, et al. Scandinavian clinical practice guideline on fluid and drug therapy in adults with acute respiratory distress syndrome. Acta Anaesthesiol Scand. 2016;60(6):697-709.
12. Napolitano LM, Park PK, Raghavendran K, Bartlett RH. Nonventilatory strategies for patients with life-threatening 2009 H1N1 influenza and severe respiratory failure. Crit Care Med. 2010;38(4 Suppl):e74-90.
13. Jacobs I, Nadkarni V, Bahr J, Berg RA, Billi JE, Bossaert L, et al. Cardiac arrest and cardiopulmonary resuscitation outcome reports: update and simplification of the Utstein templates for resuscitation registries: a statement for healthcare professionals from a task force of the International Liaison Committee on Resuscitation (American Heart Association, European Resuscitation Council, Australian Resuscitation Council, New Zealand Resuscitation Council, Heart and Stroke Foundation of Canada, InterAmerican Heart Foundation, Resuscitation Councils of Southern Africa). Circulation. 2004;110(21):3385-97.
14. Black SM, Heidersbach RS, McMullan DM, Bekker JM, Johengen MJ, Fineman JR. Inhaled nitric oxide inhibits NOS activity in lambs: potential mechanism for rebound pulmonary hypertension. Am J Physiol. 1999;277(5):H1849-56.
15. Carriedo H, Rhine W. Withdrawal of inhaled nitric oxide from nonresponders after short exposure. J Perinatol. 2003;23(7):556-8.
16. Birnie K, Verheyden V, Pagano D, Bhabra M, Tilling K, Sterne JA, et al. Predictive models for kidney disease: improving global outcomes (KDIGO) defined acute kidney injury in UK cardiac surgery. Crit Care. 2014;18(6):606.
17. Regulations concerning action and limit values for physical and chemical agents in the working environment and classified biological agents (Regulations concerning Action and Limit values), (2013).
18. Cook D, Lauzier F, Rocha MG, Sayles MJ, Finfer S. Serious adverse events in academic critical care research. CMAJ. 2008;178(9):1181-4.

19. Schlapbach LJ, Horton SB, Long DA, Beca J, Erickson S, Festa M, et al. Study protocol: NITric oxide during cardiopulmonary bypass to improve Recovery in Infants with Congenital heart defects (NITRIC trial): a randomised controlled trial. BMJ Open. 2019;9(8):e026664.
20. Uppsala Monitoring Centre. The use of the WHO-UMC system for standardised case causality assessment [cited 2016 16th September]. Available from: http://www.webcitation.org/6kYn8ezcY
21. Buskop C, Bredmose PP, Sandberg M. A 10-year retrospective study of interhospital patient transport using inhaled nitric oxide in Norway. Acta Anaesthesiol Scand. 2015;59(5):648-53.
22. Kieser M, Friede T. Simple procedures for blinded sample size adjustment that do not affect the type I error rate. Stat Med. 2003;22(23):3571-81.
